# M2 macrophage-related gene signature in chronic rhinosinusitis with nasal polyps

**DOI:** 10.3389/fimmu.2022.1047930

**Published:** 2022-11-17

**Authors:** Ying Zhu, Xiwen Sun, Shaolin Tan, Chunyu Luo, Jiayao Zhou, Shiyao Zhang, Zhipeng Li, Hai Lin, Weitian Zhang

**Affiliations:** ^1^ Department of Otolaryngology-Head and Neck Surgery, Shanghai Sixth People’s Hospital Affiliated to Shanghai Jiao Tong University School of Medicine, Shanghai, China; ^2^ Otolaryngological Institute, Shanghai Jiao Tong University, Shanghai, China; ^3^ Shanghai Key Laboratory of Sleep Disordered Breathing, Shanghai Jiao Tong University, Shanghai, China

**Keywords:** chronic rhinosinusitis with nasal polyps, M2 macrophage, immune infiltration, differentially expressed genes, hub genes, bioinformatics

## Abstract

**Background:**

Chronic rhinosinusitis with nasal polyps (CRSwNP) is a common sinonasal inflammatory disorder with high heterogeneity. Increasing evidence have indicated that the infiltration of macrophages especially M2 macrophages play pivotal roles in the pathogenesis of CRSwNP, but the underlying mechanisms remain undetermined. This study sought to identify potential biomarkers related to M2 macrophages in CRSwNP.

**Methods:**

The expression datasets of GSE136825 and GSE179265 were download from Gene Expression Omnibus (GEO) database and merged. Then, CIBERSORT and weighted gene co-expression network analysis (WGCNA) algorithms were applied to identify M2 macrophage-related gene modules. Thereafter, differentially expressed genes (DEGs) related to M2 macrophages were selected to perform functional enrichment analyses. A protein-protein interaction (PPI) network was built to identify hub genes and quantitative real-time reverse transcriptions PCR was used to verify the bioinformatics results.

**Results:**

A total of 92 DEGs associated with M2 macrophages were identified for further analysis. The results of Gene ontology (GO) and Kyoto Encyclopedia of genes and genomes (KEGG) analyses illustrated that M2 macrophage-associated DEGs primarily enriched in immune responses and extracellular matrix structure. PPI network analysis identified 18 hub genes related to M2 macrophages that might be pivotal in the pathogenesis of CRSwNP. After verification, AIF1, C1QA, C1QB, C3AR1, CCR1, CD163, CD4, CD53, CD86, CSF1R, CYBB, FCER1G, FCGR3A, IL10RA, ITGB2, LAPTM5, PLEK, TYROBP were identified as potential M2 macrophage-related biomarkers for CRSwNP.

**Conclusion:**

These findings yield new insights into the hub genes and mechanisms related to M2 macrophages in the pathogenesis of CRSwNP. Further studies of these hub genes would help better understand the disease progression and identify potential treatment targets.

## Introduction

Chronic rhinosinusitis (CRS) is a chronic heterogenous inflammatory disease of nasal cavity and paranasal sinuses with complex etiology and high recurrent rate. Typically, CRS is classified as CRS with nasal polyps (CRSwNP) and CRS without nasal polyps (CRSsNP) according to the presence or absence of nasal polyps (1). With deeper understanding about the pathogenesis of CRS at the cellular and molecular levels, more detailed classifications based on immunopathologic biomarkers are established. CRSwNP can be further classified into eosinophilic CRSwNP (ECRSwNP) and non-eosinophilic CRSwNP (non-ECRSwNP) depending on the number of eosinophils and the proportion of eosinophils in total inflammatory cells (2). ECRSwNP mostly displays a type 2 immune response with more severe symptoms and higher recurrent rate while non-ECRSwNP is manifested as type 1 or type 3 immune responses (3). Currently, disruption of nasal epithelial barrier, increasing exposure to pathogenic microbiomes, dysfunction of the immune system and tissue remodeling are considered to play crucial roles in the pathogenesis of CRSwNP (4). In addition, recent studies have demonstrated that microRNA plays a crucial role in modulating the immune and inflammatory response through regulating the expression of targeted genes in CRSwNP (5).

Macrophages are highly plastic cells which are capable of sensing and responding to the alterations of microenvironment. Generally, activated macrophages could be subdivided as classically activated (M1) macrophages and alternatively activated (M2) macrophages (6). Activated by IFN-γ, lipopolysaccharide (LPS) or granulocyte macrophage colony-stimulating factor (GM-CSF), M1 macrophages exhibits proinflammatory abilities which can stimulate Th1 immunity, produce proinflammatory mediators and maintain inflammatory responses. Nevertheless, M2 macrophages could be activated by IL-4, IL-13, IL-10, macrophage colony-stimulating factor (M-CSF) or transforming growth factor β (TGF-β) and are able to trigger anti-inflammatory responses, promote tissue remodeling and mediate Th2 immunity (7). M1 and M2 macrophages represent two extreme status of macrophage activation and the imbalance of two types of macrophages can lead to various inflammatory or metabolic disorders (8).

Previous studies have indicated that the infiltration of M1 and M2 macrophages especially M2 macrophages are significantly increased in nasal polyps from CRSwNP patients (9, 10) and the number of M2 macrophages was positively correlated with the levels of type 2 mediators including IL-5, eosinophil cationic protein (ECP) and IgE (10). The disturbance of M1/M2 macrophage homeostasis might result in the abnormal phagocytotic function of macrophages in CRSwNP with type 2 inflammation (11). In addition, although M2 macrophages were reported to be increased in nasal polyps, the number of M2 macrophages producing IL-10 was decreased in ECRSwNP, which might lead to sustained inflammation of nasal mucosa (12). Moreover, M2 macrophages are able to regulate angiogenesis and promote extracellular matrix deposition, which could eventually result in tissue remodeling (13). However, the precise roles of M2 macrophages in CRSwNP remain uncertain.

Here, we sought to explore the gene signature related to M2 macrophages through integrating public gene expression datasets of CRSwNP and performing bioinformatics analysis. Potential hub genes related to M2 macrophages and their biological functions were identified, which could provide new insights into the molecular mechanisms of CRSwNP.

## Material and methods

### Datasets acquisition and preprocessing

GSE136825 (14) and GSE179265 (15) datasets were downloaded from Gene Expression Omnibus (GEO) database (https://www.ncbi.nlm.nih.gov/geo/). GSE136825 dataset was consisted of 28 inferior turbinate tissues from healthy control subjects and 42 nasal polyp tissues from CRSwNP patients while GSE179265 dataset included seven normal uncinate process tissues and 17 nasal polyp samples from CRSwNP patients. After filtering out low expression genes, two datasets were normalized with “NormalizeBetweenArrays” function in “limma” R package and then merged (16). Next, “combat” function in “sva” R package was applied to remove batch effects between the two datasets (17). Principal component analysis (PCA) was applied with “FactoMine” and “factoextra” R packages to examine the preprocessed data.

### Gene set enrichment analysis

GSEA is an analytical approach to interpret gene expression data based on gene sets enrichment (18). The potential biological pathways involving in the pathogenesis of CRSwNP were analyzed by performing GSEA between nasal polyp tissues and normal nasal mucosal samples. There were three steps in the analytical process of GSEA including calculation of an enrichment score (ES), estimation of significance level of ES and adjustment for multiple hypothesis testing. The “Hallmark” gene sets were downloaded from “Molecular Signatures Database V7.5.1” and selected as annotations (19). Genes from merged data were pre-ranked with fold changes calculated by “limma” R package. Next, fast gene set enrichment analysis (fgsea) algorithms (20) implemented in the “Clusterprofiler” R package were used to perform GSEA with the pre-ranked gene list. The number of permutations was set to 1000. False discovery rate (FDR) < 0.05 was deemed statistically significant.

### Landscape of immune infiltration in CRSwNP

CIBERSORT is a deconvolution algorithm to analyze the cell composition of tissues based on the input reference gene sets. Here, to estimate the abundance of infiltrating immune cells, the CIBERSORT (21) approach was applied to calculate the relative proportion of immune cells in all samples. LM22, a gene signature matrix including 547 genes which could distinguish 22 types of human immune cells, was used as the annotation gene set. Thereafter, the relative proportions of immune cells were compared between two groups and the correlations among various immune cells were calculated. “Corrplot” and “ggplot2” R packages were applied to visualize the results.

### Weighted gene co-expression network analysis

To explore the relationship between gene expression and immune cell infiltration, the highly-correlated co-expressed gene modules were constructed by WGCNA (22). Low expressed genes were filtered out to remove noise and genes with coefficient value > 0.1 were identified for further analysis using the “WGCNA” R package. In brief, an adjacency matrix was built on the basis of the soft power value β and the similarity matrix calculated by Pearson correlation analysis among all genes. Then, the adjacency matrix was converted to the topological overlap matrix (TOM) and the corresponding dissimilarity (1-TOM) with a threshold soft power of 5. Next, to sort out genes with similar expression profiles into various modules, a hierarchical clustering dendrogram was further established (minModuleSize = 30, mergeCutHeight = 0.25). Finally, we calculated the module eigengene (ME) of each module and the correlations between ME and relative proportion of distinct types of macrophages estimated above. The modules significantly correlated with M2 macrophage infiltration were focused and considered as hub modules in this study.

### Screening of M2 macrophage-associated differentially expressed genes

Firstly, DEGs analysis between nasal polyp samples and healthy tissues was performed with “limma” R package (16). Genes met the screening criteria (|log2FC| > 1, *q* < 0.05) were considered as DEGs. “Pheatmap” and “ggplot2” R packages were applied to construct the heatmap and volcano plot of DEGs respectively. Then, the intersection of DEGs and genes in hub modules were selected as M2 macrophage-associated DEGs.

### Functional and pathway enrichment analyses

Gene ontology (GO) (23) functional enrichment and Kyoto Encyclopedia of genes and genomes (KEGG) (24) pathway analyses were used to detect the biological functions and pathways of DEGs related to M2 macrophages. GO annotation could be classified into three types including biological process (BP), cellular component (CC) and molecular function (MF). “Clusterprofiler” R package (25) which has integrated GO and KEGG analyses was used to determine the significantly enriched GO terms and KEGG pathways. *P* < 0.05 and *q <*0.2 were set as the cutoff criterion. In addition, “GOplot” R package (26) was used to visualize the most significant GO terms and associated genes.

### Protein-protein interaction analysis and hub genes identification

The PPI network of M2 macrophage-associated DEGs was constructed with STRING (http://string-db.org) (Version 11.5) (27). The threshold for statistical significance was set as a combined score > 0.4. Cytoscape (Version 3.9.1) (28) software was used to visualize the PPI network. Highly interconnected gene modules were detected with “MCODE” plugin of Cytoscape (degree cutoff = 2, node score cutoff = 0.2, K-core = 2, max depth = 100). Then, to identify hub genes in the PPI network, “cytoHubba” (29) plugin of Cytoscape was used to score the genes according to their network features. Here, the results of maximum neighborhood component (MNC), maximal clique centrality (MCC), edge percolated component (EPC), degree, closeness and radiality were used to select hub genes. Finally, co-expression network of hub genes and genes sharing common biological functions was built with GeneMANIA (http://www.genemania.org/) (30).

### Validation of hub genes expression in other datasets

The expression levels of identified hub genes were verified in GSE36830 (31) and GSE23552 (32). On the basis of GPL570 (Affymetrix Human Genome U133 Plus 2.0 Array), GSE36830 used 6 uncinate process tissues from control subjects and 6 nasal polyp tissues from CRSwNP patients. GSE23552 was derived from GPL5175 (Affymetrix Human Exon 1.0 ST Array) and was consisted of 13 normal nasal mucosal samples (inferior turbinate or uncinate process) and 10 nasal polyp tissues from CRSwNP patients.

### Patient recruitment

A total of 35 subjects, including 20 patients with CRSwNP and 15 healthy control subjects, were enrolled. The diagnostic criteria of CRSwNP were established according to the European Position Paper on Rhinosinusitis and Nasal Polyps 2012 guidelines. During endoscopic surgery, we collected nasal polyps from CRSwNP patients and inferior turbinates from control subjects undergoing septoplasty for deviated septum. The detailed information of participants’ characteristics is displayed in **Table 1**. This study was approved by the Ethical Committee of Shanghai Sixth People’s Hospital Affiliated to Shanghai Jiao Tong University School of Medicine, and informed consent was obtained from each subject.

**Table 1:** Characteristics of recruited subjects.

Characteristics	Control	CRSwNP	P value^*^
Total subject no. (gender)	n=15 (8M/7F)	n=20 (12M/8F)	0.693
Age (y), median (IQR)	37 (19–66)	38 (20–67)	0.633
Patients who smoked, n (%)	4 (26.6)	6 (30)	0.829
Patients with atopy, n (%)	0 (0)	5 (25)	0.036
Patients with asthma, n (%)	0 (0)	4 (20)	0.066

CRSwNP, chronic rhinosinusitis with nasal polyps; F, female; M, male; IQR, interquartile ranges; *CRSwNP vs control.

### Quantitative real-time reverse transcriptions PCR

Total RNA in nasal tissues was extracted utilizing RNeasy commercial kit (Qiagen, Chatsworth, CA, USA). Total RNA was reverse-transcribed to cDNA and quantitative real-time PCR was conducted using SYBR Premix Ex Taq kit (TaKaRa Biotechnology, Dalian, China) with specific primers (**Supplementary Table 7**). Glyceraldehyde 3-phosphate dehydrogenase (GAPDH) was used as an internal control gene. Relative mRNA levels were expressed and calculated using 2(-Delta Delta CT) methods. A normal control nasal tissue sample was designated as the calibrator.

### Statistical analysis

The bioinformatics analysis was conducted with R software (Version 4.2.0). The comparison of 22 types of immune cells infiltration in merged datasets, the expression levels of hub DEGs in GSE23552, GSE36830 and the relative mRNA levels of hub DEGs in clinical samples from enrolled subjects between CRSwNP and control groups were carried out with Mann–Whitney U test as the data did not conform to normal distribution. Correlation analysis was performed by Spearman’s correlation. *P* < 0.05 was deemed statistically significant in all cases.

## Results

### Sketch of study design and data preprocessing

**Figure 1:**
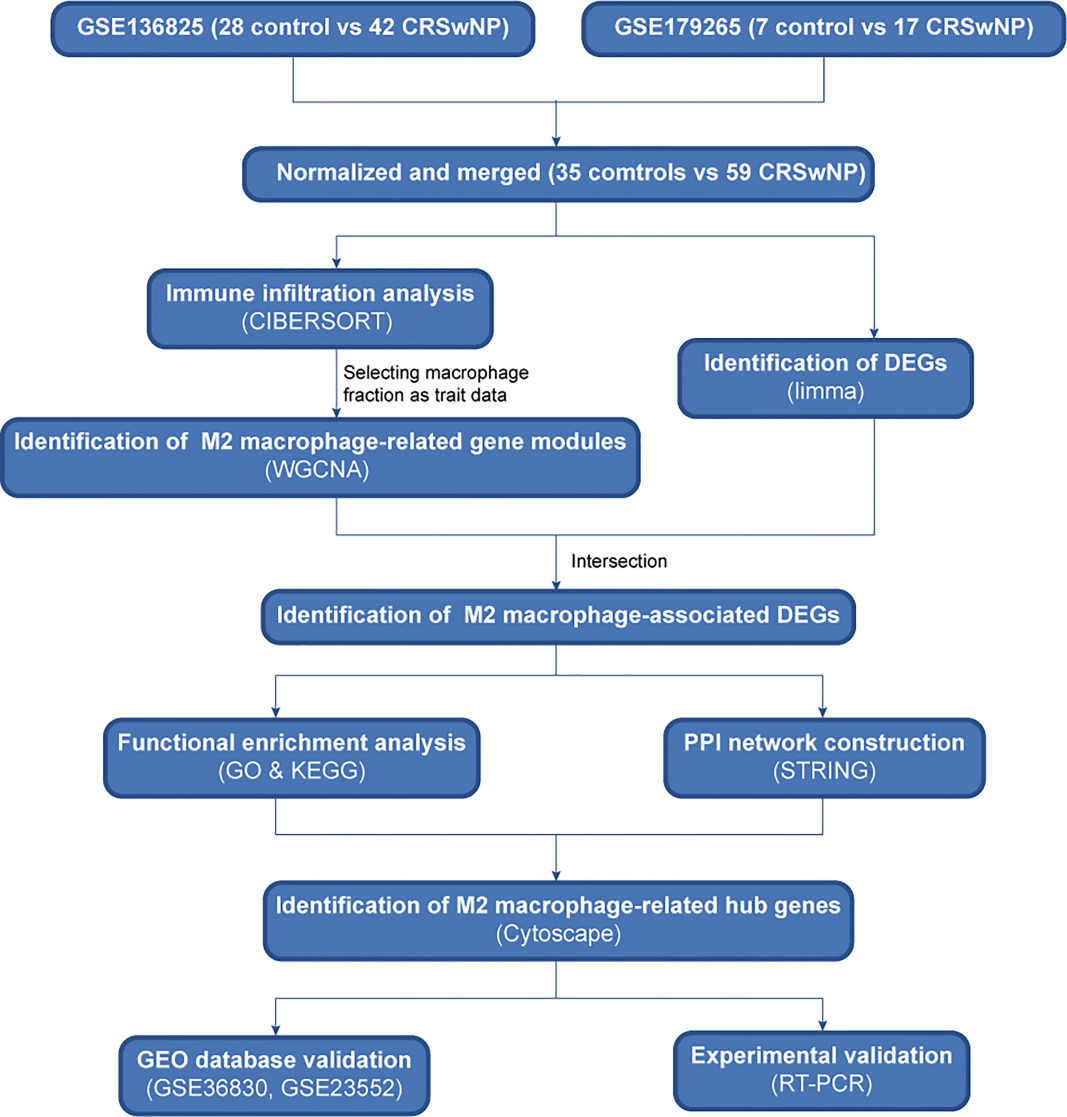
Sketch of research design. Flowchart of the study to identify hub genes related to M2 macrophages in CRSwNP. CRSwNP, chronic rhinosinusitis with nasal polyps; WGCNA, weighted gene co-expression network analysis; DEGs, differentially expressed genes; GO, Gene Ontology; KEGG, Kyoto Encyclopedia of Genes and Genomes; PPI, protein-protein interaction; RT-qPCR, real-time quantitative PCR.

The protocol of the study is illustrated in **Figure 1**. Briefly, two datasets of CRSwNP from GEO database were normalized and merged. Then, we used CIBERSORT and WGCNA algorithms to identify hub gene modules related to M2 macrophages. The intersection of differentially expressed genes and genes in hub modules was considered as M2 macrophage-associated DEGs and used to perform functional enrichment and PPI network analyses. Finally, hub genes related to M2 macrophages were identified and verified using quantitative real-time reverse transcriptions PCR method.

**Figure 2:**
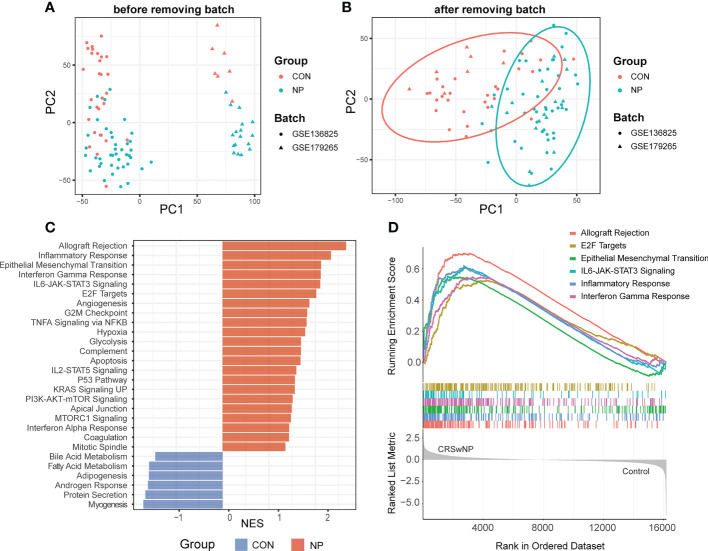
Data preprocessing and gene set enrichment analysis (GSEA). **(A)** Principal component analysis (PCA) of initial gene expression profile. **(B)** PCA of gene expression profile after removing batch effects. **(C)** Normalized enrichment scores (NES) of pathways significantly enriched in CRSwNP or control subjects. **(D)** GESA plot of the top 6 enriched pathways in CRSwNP. CON, controls; NP, nasal polyps.

To reduce the ratio of false positives in a single dataset, we integrated two datasets from the same platform (GSE136825 and GSE179265). After background correlation and normalization, the batch effects between two datasets were eliminated (**Figures 2A, B**). Ultimately, 94 samples (35 controls and 59 CRSwNP) and 16162 intersected genes were screened for subsequent analysis.

### Identification of pathways involved in CRSwNP by GSEA

To explore crucial pathways involved in the pathogenesis of CRSwNP, a preliminary functional analysis was performed between two groups. GSEA based on “Hallmarks” database revealed the biological functions associated with CRSwNP including 22 up-regulated pathways and 6 down-regulated pathways (**Figure 2C**). Notably, allograft rejection, epithelial mesenchymal transition (EMT), E2F targets and several immune functions such as inflammation response, IFN-γ response and IL6-JAK-STAT3 signaling were significantly enriched in CRSwNP (**Figure 2D**). Consistent with our results, previous studies have indicated that EMT, inflammation response, IFN-γ response, IL6-JAK-STAT3 signaling play crucial roles in the pathogenesis of CRSwNP (1, 33). As immune responses play a pivotal role in CRSwNP, we estimated the immune cell infiltrations of nasal polyps in the following analysis.

### Immune cells infiltration of nasal polyp and healthy nasal mucosal samples

**Figure 3:**
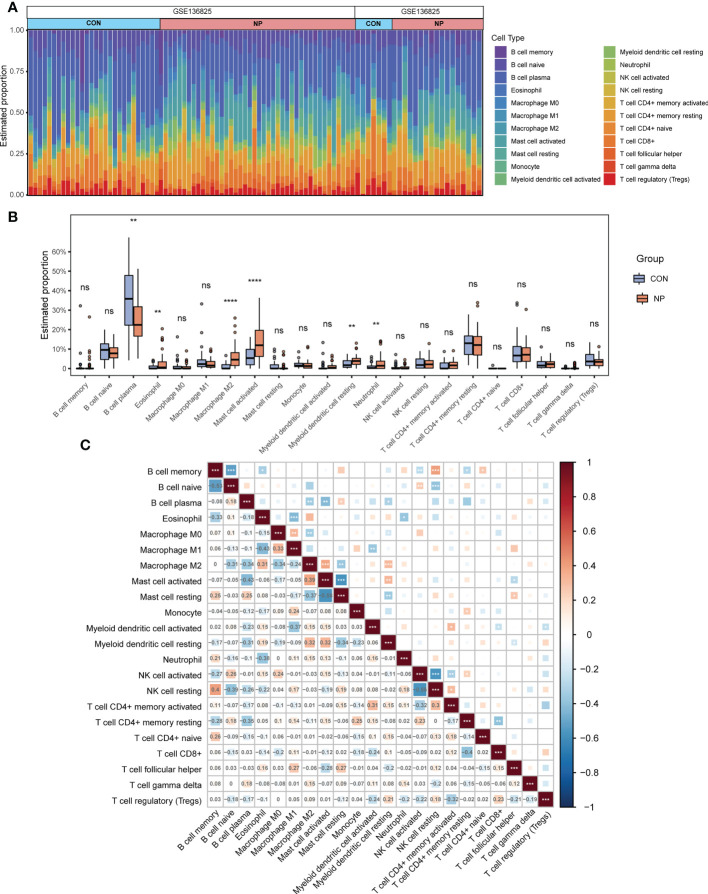
Immune cells infiltration in nasal polyps and normal nasal mucosal samples. **(A)** Estimated proportion of 22 types of immune cells in all eligible samples. **(B)** Comparison of immune cells infiltration between CRSwNP group and control group. **(C)** Correlations among 22 types of infiltrating immune cells (red indicates a positive correlation, blue indicates a negative correlation, and the depth of the color indicates the degree of correlation). ns: not significant. *P < 0.05, **P < 0.01, ***P < 0.001, ****P < 0.0001.

To identify immune infiltration landscape in all samples from the merged datasets, we applied the CIBERSORT algorithm to calculate the relative abundance of 22 types of immune cells. The estimated percentages of immune cells in 94 samples are displayed in **Figure 3A**. Next, the infiltration of different immune cells between two groups was compared. The proportions of eosinophil, M2 macrophage, activated mast cell, resting myeloid dendritic cell and neutrophil were significantly increased while the relative proportion of plasma cell was decreased in nasal polyp tissues compared with control tissues (**Figure 3B**). **Figure 3C** describes the correlation between different types of immune cells. M2 macrophages were positively correlated with activated mast cells (r = 0.39) and resting myeloid dendritic cells (r = 0.32) and negatively correlated with plasma cells (r = -0.34), resting mast cells (r = -0.37) and M0 macrophages (r = -0.34). Therefore, the infiltration of M2 macrophages significantly increased and might play an important role in the pathogenesis of CRSwNP. To further explore macrophage-associated genes, the estimated proportion of 3 distinct types of macrophages were sorted out as trait data for subsequent analysis (**Supplementary Table 1**).

### Construction of co-expression network and identification of hub modules

**Figure 4:**
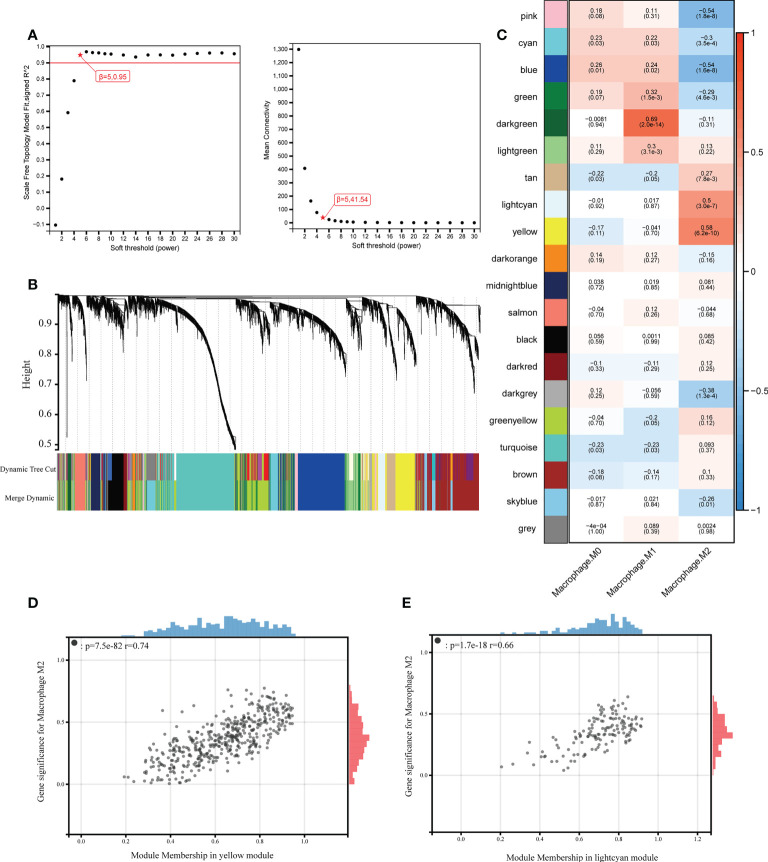
Construction of weighted gene co-expression network. **(A)** Selection of the soft threshold when the index of scale-free topologies reaches 0.90 and analysis of the mean connectivity of 1–20 soft threshold power. **(B)** Establishment of co-expressed gene modules based on hierarchical clustering algorithm. **(C)** Correlations between module eigengene and macrophages infiltration. The number on the top of each square indicates the correlation coefficients between macrophages infiltration and corresponding modules, along with the P values shown in the brackets below (red indicates a positive correlation, blue indicates a negative correlation, and the depth of the color indicates the degree of correlation). **(D)** Correlation of module membership in yellow modules and gene significance for M2 macrophages. **(E)** Correlation of module membership in light cyan modules and gene significance for M2 macrophages.

To investigate the genes correlated with M2 macrophages infiltration, we performed WGCNA which could divide genes into different modules according to their connections. Gene co-expression network was constructed with 6508 eligible genes through “WGCNA” R package. To establish a scale-independent topological network, the soft threshold power (β) was set as 5 (scale-free R2 = 0.9) (**Figure 4A**). Genes with analogous expression patterns were classified into same gene modules based on hierarchical clustering method and a total of 20 modules were identified after highly correlated modules were fused (**Figure 4B**). Module eigengene, representation of expression profile of all genes in the module, was applied to calculate the correlations between different gene modules and macrophages infiltration. It turned out that the yellow module (r = 0.58, p = 6.2e-10) and the light cyan module (r = 0.50, p = 3.0e-7) were positively correlated with M2 macrophages infiltration (**Figure 4C**). The correlations of gene module membership in yellow and light cyan module and gene significance for M2 macrophages are shown in **Figures 4D, E**. As the abundance of M2 macrophages was significantly increased in nasal polyp tissues, yellow and light cyan modules with a total of 596 genes (457 in yellow and 139 in light cyan) were considered as hub modules for further analysis.

### Identification of M2 macrophage-associated DEGs

**Figure 5:**
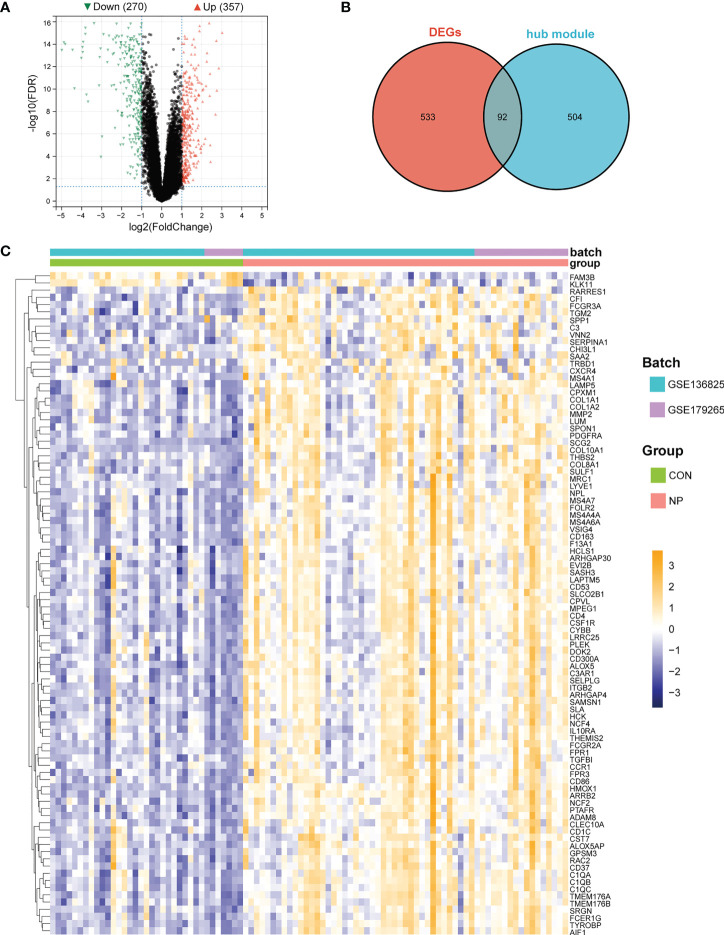
Identification of M2 macrophage-associated DEGs. **(A)** Volcano plot of DEGs between nasal polyps and normal nasal mucosa samples. **(B)** Venn diagram of intersection genes between DEGs and genes in M2 macrophages-related gene modules. **(C)** Heatmap of M2 macrophage-associated DEGs. The color bar indicates the relative expression levels of genes in each sample (yellow indicates relative high expression, purple indicates relative low expression and the depth of the color indicates the level of high or low gene expression).

Next, we aimed to identify genes with significantly altered expression in M2 macrophage-related gene modules. We firstly used “limma” algorithm to screen for DEGs in CRSwNP. A total of 627 DEGs consisting of 357 up-regulated genes and 270 down-regulated genes were identified between nasal polyps and normal tissues (**Figure 5A**). Next, 92 genes intersected between 627 DEGs and 596 genes in hub modules (ninety up-regulated and two down-regulated) were identified as M2 macrophage-associated DEGs (**Figure 5B**). The relative expressions of M2 macrophage-associated DEGs were visualized with a heatmap (**Figure 5C**) and details of these DEGs can be seen in the supplementary materials (**Supplementary Table 2**). These M2 macrophage-associated DEGs were used to perform subsequent functional enrichment analysis.

### Functional enrichment and pathway analyses of M2 macrophage-associated DEGs

**Figure 6:**
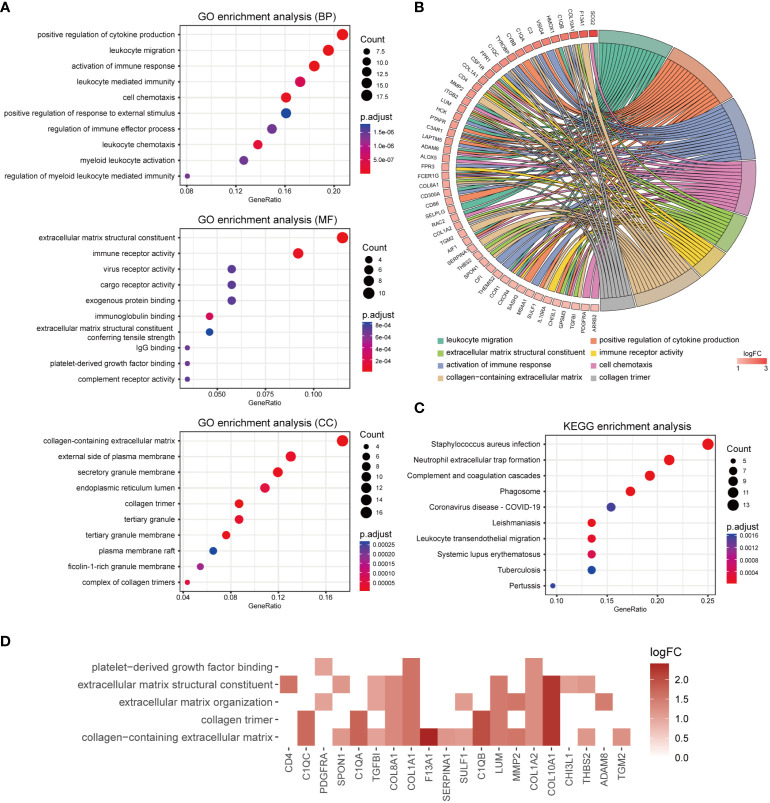
Functional enrichment analyses of M2 macrophage-associate DEGs. **(A)** Bubble chart showing enriched gene ontology (GO) terms for M2 macrophage-associated DEGs. **(B)** Chord diagram of the most significant enriched GO terms for M2 macrophage-associated DEGs. Gene symbols and their fold changes are displayed on the left side, along with differently colored lines representing the involvement of genes in various GO terms. **(C)** Bubble chart showing enriched Kyoto Encyclopedia of genes and genomes (KEGG) terms for M2 macrophage-associated DEGs. **(D)** Heatmap of genes involving in tissue remodeling associated GO terms.

To investigate the potential biological functions of M2 macrophage-associated DEGs, GO and KEGG pathway enrichment analyses were performed (**Supplementary Tables 3**, **4**). The GO enrichment results indicated that M2 macrophage-associated DEGs were primarily related to immune responses and extracellular matrix. To be specific, the most enriched BP terms were positive regulation of cytokine production, leukocyte migration and activation of immune response. The most enriched MF terms were extracellular matrix structural constituent, immune receptor activity and virus receptor activity. The most enriched CC terms were collagen-containing extracellular matrix, external side of plasma membrane and secretory granule membrane (**Figure 6A**). The top enriched GO terms and relevant genes were visualized in a chord graph (**Figure 6B**). According to KEGG pathways analysis, M2 macrophage-associated DEGs were mainly related to *Staphylococcus aureus* infection, neutrophil extracellular trap formation, complement and coagulation cascades and phagosome (**Figure 6C**). As M2 macrophages were involved in tissue remodeling, we investigated the expression of genes in relevant GO terms including platelet derived growth factor binding, extracellular matrix structural constituent, extracellular matrix organization, collagen trimer and collagen-containing extracellular matrix. The results showed that 20 genes related to M2 macrophages including CD4, SULF1, SPON1, THBS2, TGM2, TGFBI, SERPINA1, MMP2, LUM, F13A1, COL10A1, COL8A1, COL1A2, COL1A1, C1QC, C1QB, C1QA, CHI3L1, ADAM8 and PDGFRA were related to tissue remodeling (**Figure 6D**). The primary functions of these genes are displayed in **Supplementary Table 5**.

### PPI network of M2 macrophage-associated DEGs and identification of hub genes

**Figure 7:**
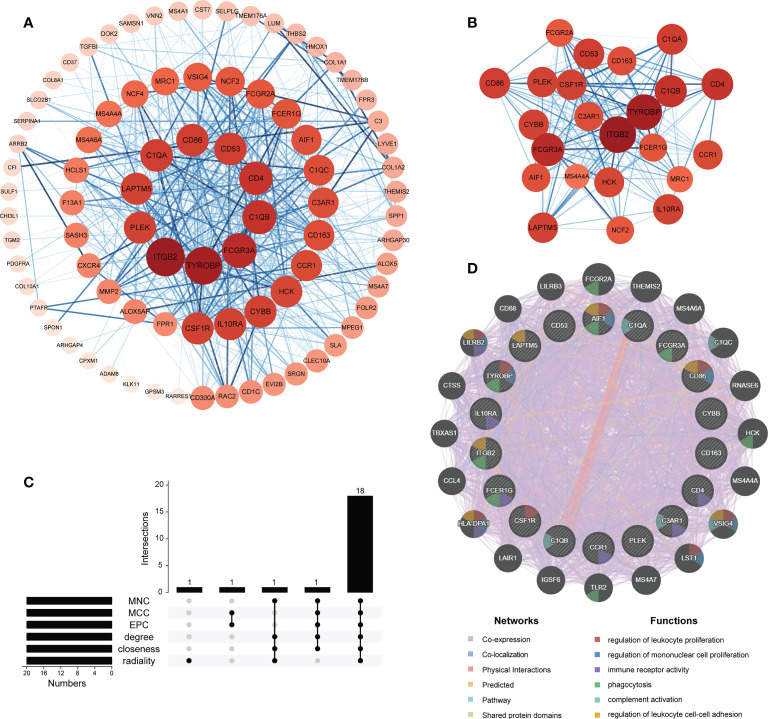
Protein-protein interaction (PPI) analyses of M2 macrophage-associate DEGs. **(A)** PPI network of M2 macrophage-associate DEGs. **(B)** The most significant gene clustering module identified by MCODE plugin of Cytoscape software. **(C)** Venn diagram of 6 similar algorithms in cytoHubba plugin which screened out 18 overlapping hub genes. **(D)** Network of hub genes and their co-expressed genes identified by GeneMANIA.

To further identify hub genes among 92 M2 macrophage-related DEGs, a PPI network was constructed with Cytoscape software containing 91 nodes and 705 edges (**Figure 7A**). The network was ranked by the degree parameter calculated with cytoHubba plugin of Cytoscape software. Genes with higher degrees indicate more significant roles in the network. Thereafter, the most closely connected gene module containing 23 common genes and 225 interaction pairs was identified by MCODE plugin of Cytoscape which was consisted of genes with the most similar biologic functions (**Figure 7B**). Next, to screen for the most important genes in this network, we combined the results of 6 similar algorithms in cytoHubba. The top 20 hub genes calculated by 6 algorithms were intersected and 18 hub genes were obtained eventually (**Supplementary Table 6**, **Figure 7C**). The brief introductions of 18 hub genes including AIF1, C1QA, C1QB, C3AR1, CCR1, CD163, CD4, CD53, CD86, CSF1R, CYBB, FCER1G, FCGR3A, IL10RA, ITGB2, LAPTM5, PLEK, TYROBP are listed in **Table 2**. Among these genes, PLEK was reported to be involved in CRSwNP but not correlated with M2 macrophages while AIF1, CD53 and LAPTM5 were related to M2 macrophages but not explored in nasal polyps. The rest of hub genes have been reported to be correlated with M2 macrophages and participate in the pathogenesis of CRSwNP respectively, but whether these genes primarily interact with M2 macrophages in nasal polyps remains unclear. Next, to further explore the interaction of hub genes, a co-expression network and its biological functions were analyzed with GeneMANIA database. A network containing 38 genes (18 hub genes and 20 related genes) and 2658 links was identified (91.17% co-expression, 4.26% co-localization, 3.11% physical interactions, 0.96% predicted, 0.42% pathway and 0.08% shared protein domain). The functions of hub genes were mainly enriched in regulation of leukocyte and mononuclear cell proliferation, immune receptor activity, phagocytosis, complement activation and regulation of leukocyte cell-cell adhesion (**Figure 7D**).

**Table 2:** Detail information of the M2 macrophage-associated hub genes.

Genes	Description	Function
**AIF1**	allograft inflammatory factor 1	encodes a protein that binds actin and calcium, promots macrophage activation and growth of vascular smooth muscle cells and T-lymphocytes
**C1QA**	complement C1q A chain	component of the serum complement system
**C1QB**	complement C1q B chain	component of the serum complement system
**C3AR1**	complement C3a receptor 1	an orphan G protein-coupled receptor for C3a, activates chemotaxis, granule enzyme release, superoxide anion production and bacterial opsonization
**CCR1**	C-C motif chemokine receptor 1	a member of the beta chemokine receptor family, receptor of MIP-1 alpha, RANTES, MCP-3 and MPIF-1
**CD163**	CD163 molecule	a member of the scavenger receptor cysteine-rich (SRCR) superfamily, exclusively expressed in monocytes and macrophages
**CD4**	CD4 molecule	a coreceptor with the T-cell receptor on the T lymphocyte to recognize antigens displayed by an antigen presenting cell in the context of class II MHC molecules
**CD53**	CD53 molecule	a member of the transmembrane 4 superfamily, mediate signal transduction events that play a role in the regulation of cell development, activation, growth and motility
**CD86**	CD86 molecule	a type I membrane protein that is a member of the immunoglobulin superfamily, expressed by antigen-presenting cells
**CSF1R**	colony stimulating factor 1 receptor	the receptor for colony stimulating factor 1, a cytokine which controls the production, differentiation, and function of macrophages
**CYBB**	cytochrome b-245 beta chain	composition of cytochrome b, a primary component of the microbicidal oxidase system of phagocytes
**FCER1G**	Fc epsilon receptor Ig	gamma chain of high affinity IgE receptor
**FCGR3A**	Fc gamma receptor IIIa	a receptor for the Fc portion of IgG, involved in the removal of antigen-antibody complexes from the circulation
**IL10RA**	interleukin 10 receptor subunit alpha	a receptor for interleukin 10, mediate the immunosuppressive signal of interleukin 10, inhibits the synthesis of proinflammatory cytokines
**ITGB2**	integrin subunit beta 2	combines with multiple different alpha chains to form different integrin heterodimers, integral cell-surface proteins that participate in cell adhesion as well as cell-surface mediated signalling
**LAPTM5**	lysosomal protein transmembrane 5	a transmembrane receptor that is associated with lysosomes
**PLEK**	pleckstrin	enables phosphatidylinositol-3,4-bisphosphate binding activity; protein homodimerization activity; and protein kinase C binding activity
**TYROBP**	transmembrane immune signaling adaptor TYROBP	a transmembrane signaling polypeptide, associate with the killer-cell inhibitory receptor (KIR) family of membrane glycoproteins and may act as an activating signal transduction element

### Validation of M2 macrophage-associated hub genes in CRSwNP

**Figure 8:**
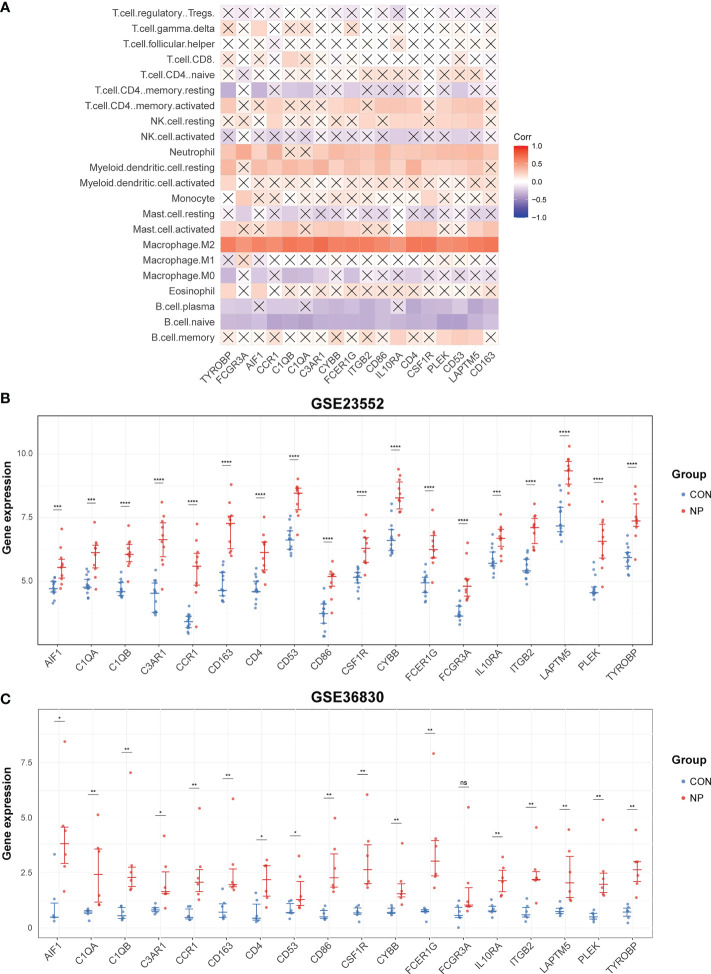
Validation of hub genes. **(A)** Correlation analysis of 22 immune cells and hub genes. The cross-out line represents that the correlation is not significant (*P* > 0.05). **(B)** The expression levels of hub genes in GSE23552. **(C)** The expression levels of hub genes in GSE36830. ns: not significant. *P < 0.05, **P < 0.01, ***P < 0.001, ****P < 0.0001.

To verify the interaction between hub genes and macrophages, we calculated the correlation of hub genes and the relative proportion of 22 immune cells in merged datasets of GSE136825 and GSE179265. As a result, all 18 hub genes were most significantly correlated with M2 macrophages among 22 types of immune cells (**Figure 8A**; **Supplementary Figure 1**).

Then, to validate the expression of hub genes in nasal polyps, we selected two other datasets containing gene expression data of CRSwNP and control subjects. Of note, all hub genes were significantly up-regulated in nasal polyp tissues compared with control tissues in GSE23552 dataset (**Figure 8B**). However, in GSE36830 dataset, only 17 hub genes were significantly increased in nasal polyps. Although not reaching statistical significance, the expression of FCGR3A did show an upward trend in nasal polyp tissues comparing with normal tissues (*p* = 0.093) (**Figure 8C**).

### Expression levels of identified hub genes in nasal polyp tissues and normal nasal mucosal samples

**Figure 9:**
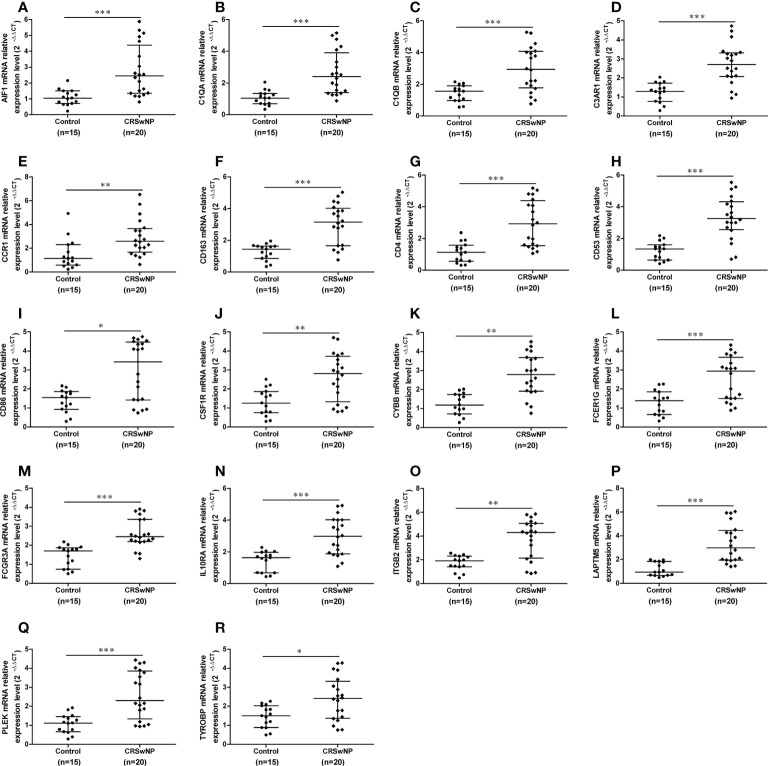
The gene expression levels of hub genes in nasal polyps and control samples. The relative expression levels of the AIF1 **(A)**, C1QA **(B)**, C1QB **(C)**, C3AR1 **(D)**, CCR1 **(E)**, CD163 **(F)**, CD4 **(G)**, CD53 **(H)**, CD86 **(I)**, CSF1R **(J)**, CYBB **(K)**, FCER1G **(L)**, FCGR3A **(M)**, IL10RA **(N)**, ITGB2 **(O)**, LAPTM5 **(P)**, PLEK **(Q)** and TYROBP **(R)** in CRSwNP (n = 20) and control subjects (n = 15). GAPDH was used as a reference. *P < 0.05, **P < 0.01, ***P < 0.001.

The mRNA expression levels of 18 hub genes in nasal tissues from 20 patients with CRSwNP and 15 controls were detected with quantitative real-time reverse transcriptions PCR to further validate the reliability of hub genes. The results indicated that the expression of AIF1, C1QA, C1QB, C3AR1, CCR1, CD163, CD4, CD53, CD86, CSF1R, CYBB, FCER1G, FCGR3A, IL10RA, ITGB2, LAPTM5, PLEK and TYROBP were all significantly increased in nasal polyps from CRSwNP patients in comparison to control tissues (**Figures 9A-R**), which are consistent with the above results. In conclusion, our study has successfully identified 18 hub genes related to M2 macrophages which might collaborate with M2 macrophages to promote the formation of CRSwNP.

## Discussion

Previous studies have indicated that the infiltration of macrophages especially M2 macrophages was significantly increased in nasal polyp tissues (9). The number of M2 macrophages was positively correlated with type 2 inflammatory mediators and contributed to the severity of chronic rhinosinusitis (10). In addition, the expression levels of several genes related to tissue remodeling such as matrix metalloproteinases (MMPs) were robustly elevated in CRSwNP (34). However, the precise mechanisms of M2 macrophages involved in the pathogenesis of CRSwNP have not been completely understood. In the present study, we investigated the hub genes related to M2 macrophages in CRSwNP by integrating two GEO databases. The infiltration of 22 types of immune cells was estimated with CIBERSOTR algorithm and the proportion of M2 macrophages was significantly elevated in CRSwNP. Next, by combining the results of DEGs and WGCNA, 92 DEGs associated with M2 macrophages were identified and these DEGs were primarily enriched in immune responses and extracellular matrix structure. Eventually, hub genes were selected by PPI analysis and then verified with real-time reverse transcriptions PCR, which might be crucial biomarkers related to M2 macrophages in CRSwNP. Although the datasets were previously published, former studies didn’t focus on the relationship between M2 macrophage infiltration and CRSwNP when analyzing the gene expression data. In addition, the precise roles of M2 macrophages in CRSwNP remain uncertain. Therefore, our study aimed to identify potential genes related to M2 macrophages in CRSwNP through bioinformatics analysis.

M2 macrophages participates in the tissue remodeling of CRSwNP. As one of the crucial regulators of tissue remodeling, the abnormally increased M2 macrophages can promote the over-production of growth factors (13). Numerous studies have confirmed the elevated levels of various growth factors such as TGF-β, VEGF and PDGF in CRSwNP (35–37). The growth factors secreted by increased M2 macrophages contribute to the proliferation of fibroblasts and the destruction of extracellular matrix which might aggravate the tissue remodeling in CRSwNP. A recent study suggested that cold-inducible RNA-binding protein (CIRP), a newly identified damage-associated molecular pattern (DAMP), could increase the number of macrophages producing MMPs and VEGF-A, thereby contributing to tissue edema and the formation of nasal polyps (34). Moreover, M2 macrophages are associated with the regulation of the vascular permeability and the coagulation system (38, 39). A previous study indicated that factor XIII-A (FXIII-A) produced by M2 macrophages might cause the aberrant deposition of fibrin and aggravate tissue remodeling in CRSwNP (40). Consistent with previous studies, our results suggest that M2 macrophage-associated DEGs were significantly enriched in tissue remodeling related functions including PDGF binding, extracellular matrix structural constituent, extracellular matrix organization, collagen trimer, collagen-containing extracellular matrix complement and coagulation cascades pathways. Of note, genes encoding growth factors (TGFBI and PDGFRA), collagens (COL10A1, COL8A1, COL1A2 and COL1A1), complement (C1QA, C1QB and C1QC), matrix metallopeptidase (MMP2) were included in the M2 macrophage-related DEGs. Previous studies have indicated that macrophages were one of the primary cells producing MMPs which could regulate the homeostasis of extracellular matrix deposition and degradation (41). Meanwhile, M2 macrophage could secrete TGF-β to promote the synthesis of collagens (13). There are also evidences indicating that M2 macrophages could interact with the complement system to mediate tissue remodeling (42). Thus, these findings were consistent with our bioinformatics analysis, demonstrating the crucial role of M2 macrophages in the tissue remodeling of CRSwNP.

M2 macrophages are also involved in the regulation of anti-inflammatory responses and primarily mediate type 2 immunity. CD163, a member of the scavenger receptor cysteine-rich (SRCR) superfamily, is one of the distinct surface markers of M2 macrophages. Previous studies have demonstrated that the number of CD163^+^ M2 macrophages was robustly increased in both ECRSwNP and non-ECRSwNP (12). CD86 is expressed by antigen-presenting cells including monocytes, dendritic cells (DCs) and activated B cells which is a costimulatory factor for T cells activation when combining with CD28. Elevated numbers of CD86^+^ cells were found in nasal polyps, leading to enhanced Th2 inflammation (43). CCR1 is a member of beta chemokine receptor family which could recruit inflammatory cells after binding with macrophage associated proteins including monocyte chemotactic protein 1 (MCP-1) and macrophage inflammatory protein 1 (MIP-1) (44). A recent study suggested that overproduced chemokine CCL23 is involved in the pathogenesis of CRSwNP possibly through recruiting CCR1^+^ inflammatory cells and enhancing local immune responses (45). IL-10R is the receptor of IL-10. IL-10, a typical Th2 cytokine, is capable of regulating M2 macrophages polarization and plays an essential role in regulating inflammatory responses of CRSwNP (46). However, a recent study indicated that the IL-10 production ability of M2 macrophages was impaired in ECRSwNP, though the number of M2 macrophages was increased (12). Thus, further studies are needed to illustrate the interaction of IL-10 and M2 macrophages in CRSwNP. FCER1G which encodes the gamma chain of high-affinity IgE receptor (FcϵRI), was primarily expressed by mast cells in nasal polyp tissues (47). Previous studied have indicated that the expression of IgE and its high-affinity receptor FcϵRI was related to the presence of mast cells (48) which was consistent with our findings that there was a weak positive correlation between FCER1G expression and mast cells infiltration. As the infiltration of M2 macrophages was positively correlated with the expression levels IgE and its high-affinity receptor in nasal polyps (10), it can be surmised that the interaction of M2 macrophages and IgE contributes to the activation of mast cells in CRSwNP. FCGR3A which encodes the Fc gamma receptor III (FcγRIII), could damage nasal epithelial cells and their ability to resist invading pathogens in CRSwNP patients (49). Meanwhile, a previous study has suggested that activated M2 macrophages could express high levels of FcγR in colorectal carcinomas (50). Thus, increased infiltration of M2 macrophages might express high levels of FcγRIII and impair nasal epithelial cells in CRSwNP.

The complement system is able to respond to microbial invaders and endogenetic threats in a rapid and extensive way. It comprises of a network of plasma and membrane proteins that play vital roles in maintaining immune homeostasis and contributing to immune surveillance (51). Our study demonstrated that complement and coagulation cascades pathways were significantly enriched in CRSwNP. C1QA and C1QB are the components of complement C1q, the expression of which was significantly increased in nasal polyps according to a previous study (52). Similarly, the expression of C3AR1, receptor of complement C3a, was also increased in CRSwNP and correlated with disease severity. Inhibition of C3a receptor attenuated CRS in an animal model, which indicated that C3a receptor might be a potential therapeutic target for CRSwNP (53). It has been demonstrated that C1q could regulate the differentiation of macrophages to an anti-inflammatory M2 type (54). Additionally, high levels of C3a could interact with M2 macrophages and contribute to angiogenesis in tumor microenvironment (42). It would be interesting to investigate the deeper mechanisms of the interaction between complement system and M2 macrophages in CRSwNP in future studies.

Previous bioinformatics analyses have identified CYBB, CSF1R, PLEK, ITGB2 and TYROBP as hub genes in the pathogenesis of CRSwNP (55, 56). Our study further suggests that these genes might contribute to CRSwNP through interacting with M2 macrophages. CYBB, also known as NADPH oxidase 2 (NOX2), is a main composition of the microbicidal oxidase system in phagocytes. NOX2 modulates the defense against microbes mainly through the generation of autophagy in macrophages and neutrophil extracellular traps (NETs) (57). However, although the up-regulation of NOX2 was discovered in CRSwNP, Zheng et al. found that p67^phox^, one of the subunits of NOX2, was expressed by neutrophils and eosinophils rather than macrophages (58). Therefore, whether M2 macrophages modulate the expression of NOX2 in an indirect way needs further investigation. CSF1R is the receptor of CSF1 (also known as M-CSF) which performs as an essential regulator of macrophage activation, polarization and proliferation (59). Macrophages undergoing M-CSF stimulation are activated to a phenotype that could regulate angiogenesis and tissue remodeling (60). PLEK is a major substrate of protein kinase C (PKC) in the platelet and regulates the secretion of the platelet (61). A knockout mice model indicated that platelets lacking PLEK has a limited ability of granule fusion to the cell membrane which led to exocytosis deficiency (62). Whether M2 macrophages participates in the regulation of PLEK expression remains unknown. ITGB2 is a subunit of integrins extensively expressed in leukocytes which could conduct the extracellular signals and modulate cell responses such as cell adhesion, proliferation and differentiation (63). A recent study showed that ITGB2 was up-regulated in CRSwNP and might serve as an inflammatory mediator (64). TYROBP is a transmembrane polypeptide that regulate signal transduction in dendritic cells, macrophages and microglia. Increasing evidence has demonstrated that TYROBP strengthen the phagocytotic ability of microglia and inhibited the immune responses in Alzheimer’s disease (65). Besides, several researches have indicated the association of TYROBP and macrophage polarization in cancers through bioinformatics analysis (66, 67). LAPTM5, a transmembrane receptor of lysosomes, is primarily expressed on immune cells and could interact with the ubiquitin ligands. LAPTM5 was reported to positively modulate inflammatory responses of macrophages but negatively regulate the receptor of T cells and B cells (68). Although the expression of CSF1R, PLEK, ITGB2, TYROBP and LAPTM5 and their relationship with M2 macrophages in CRSwNP has not been explored yet, these genes are potential M2 macrophage-related biomarkers involving in the pathogenesis of CRSwNP.

Several limitations should be noted in the present study. Firstly, we focused on M2 macrophages-associated hub genes in CRSwNP regardless of its endotypes. The gene expression data of CRSwNP used in this study didn’t include detailed endotypes information of the nasal polyp samples. Besides, current datasets containing different CRSwNP endotypes were limited. Therefore, it would be necessary to investigate the genes related to M2 macrophages among CRSwNP with different endotypes. In addition, we identified 18 hub genes related to M2 macrophages in CRSwNP only on mRNA levels. The precise roles of these hub genes in the pathogenesis of CRSwNP need to be further elucidated. Lastly, the relative proportions of other immune cells were also changed in nasal polyp samples which could be explored in future studies.

## Conclusion

To sum up, by integrating gene expression data of CRSwNP and preforming CIBERSORT and WGCNA algorithms, our study illustrated the underlying mechanisms of M2 macrophage in the development of CRSwNP. Our findings suggested that M2 macrophages regulate the pathogenesis of CRSwNP through complex immune responses and tissue remodeling. Then, 18 genes were identified as M2 macrophages-associated hub genes of CRSwNP which yield new insight into the pathogenesis of CRSwNP at the cellular and molecular level; nevertheless, future studies are needed to explore the interaction of these genes and M2 macrophages in CRSwNP.

## Data availability statement

The datasets presented in this study can be found in online repositories. The names of the repository/repositories and accession number(s) can be found below: https://www.ncbi.nlm.nih.gov/geo/, GSE136825, GSE179265, GSE23552, GSE36830.

## Ethics statement

The studies involving human participants were reviewed and approved by Ethical Committee of Shanghai Sixth People’s Hospital Affiliated to Shanghai Jiao Tong University School of Medicine. The patients/participants provided their written informed consent to participate in this study.

## Author contributions

YZ and HL designed the study. YZ drafted the manuscript. HL, YZ, XS, CL, ST, JZ, ZL and SZ contributed to the enrollment of subjects and data collection. HL performed the experiments. YZ performed the analysis. HL and WZ contributed to the interpretation of the results, and reviewed and edited the manuscript. All authors contributed to the article and approved the submitted version.

## Funding

This work was supported by grants from the National Natural Science Foundation of China (No. 82071014, No.82271137, No. 81870700 and No. 82000951), Shanghai Natural Science Foundation of China (No. 16ZR1426100), Science and Technology Innovation Action Plan of Science and Technology Commission of Shanghai Municipality (No. 19411950700), Clinical Science and Technology Innovation Project of Shanghai Shen‐Kang Hospital Management Center (SHDC12020129) and the Key Laboratory Funding of Science and Technology Commission of Shanghai Municipality (No. 18DZ2260200).

## Acknowledgments

We thank the researchers who contributed to the GEO datasets, as well as the patients and healthy volunteers who participated in this study.

## Conflict of interest

The authors declare that the research was conducted in the absence of any commercial or financial relationships that could be construed as a potential conflict of interest.

## Publisher’s note

All claims expressed in this article are solely those of the authors and do not necessarily represent those of their affiliated organizations, or those of the publisher, the editors and the reviewers. Any product that may be evaluated in this article, or claim that may be made by its manufacturer, is not guaranteed or endorsed by the publisher.
