# Supplementary material for: M2 macrophage-related gene signature in chronic rhinosinusitis with nasal polyps
Source: Front Immunol. 2022 Nov 17;13:1047930. doi: 10.3389/fimmu.2022.1047930 (PMC9712459; doi:10.3389/fimmu.2022.1047930)
Supplement: Supplementary file 1 [file DataSheet_1.docx]

Supplementary Material

# Supplementary Figures and Tables

## Supplementary Figures


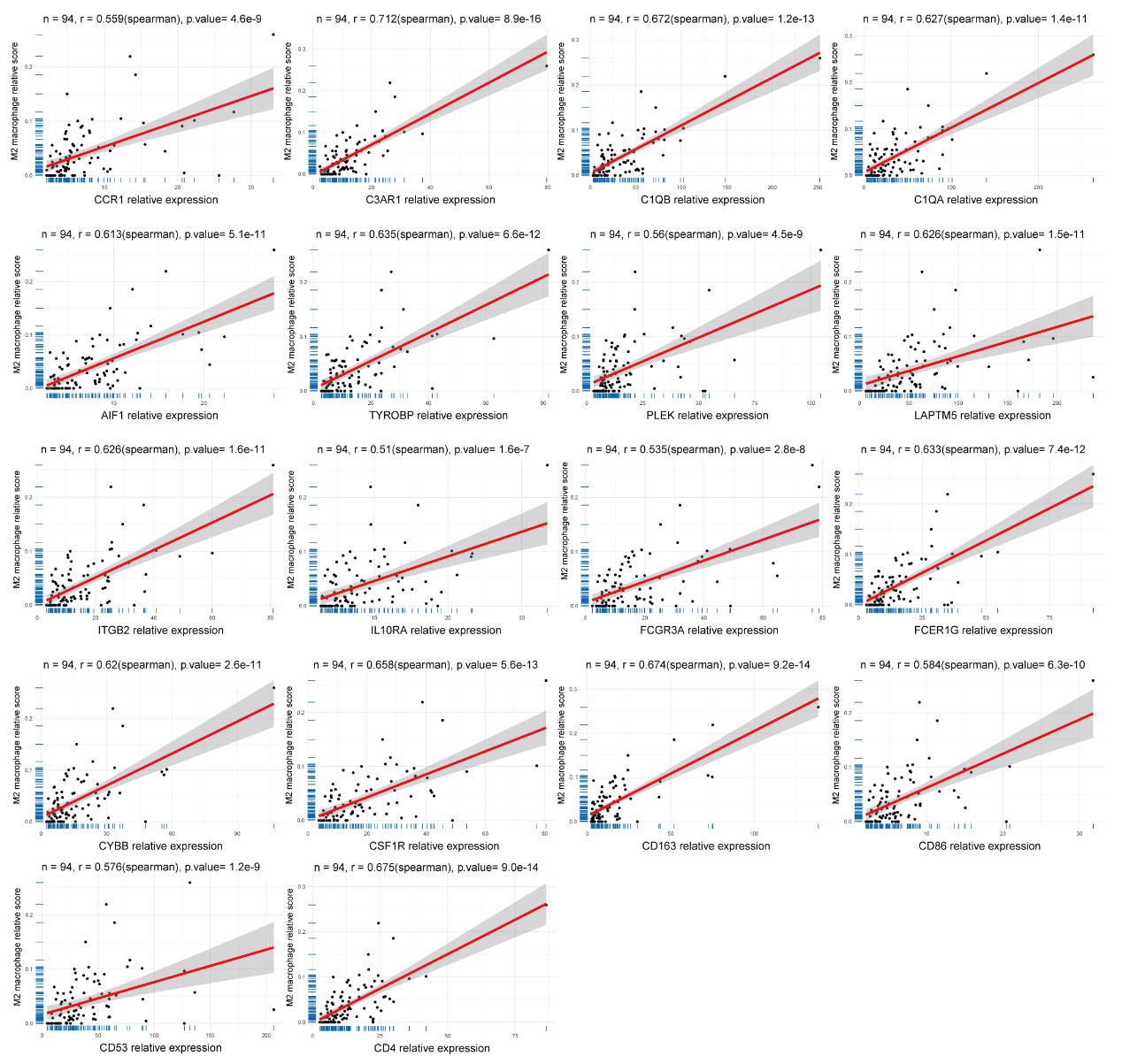


**Supplementary Figure 1.** Correlations of hub genes and M2 macrophages infiltration. Correlation analyses of M2 macrophages infiltration and AIF1, C1QA, C1QB, C3AR1, CCR1, CD163, CD4, CD53, CD86, CSF1R, CYBB, FCER1G, FCGR3A, IL10RA, ITGB2, LAPTM5, PLEK, TYROBP genes with Spearman’s method.

## Supplementary Tables

**Supplementary Table 1**. Estimated proportions of macrophages in all samples.

| **Sample** | **Group** | **Macrophage M0** | **Macrophage M1** | **Macrophage M2** |
| --- | --- | --- | --- | --- |
| GSM4058752 | NP | 0.022233 | 0.034512 | 0.057101 |
| GSM4058753 | NP | 0 | 0.041804 | 0.022993 |
| GSM4058754 | NP | 0.090435 | 0.034261 | 0.090664 |
| GSM4058755 | NP | 0.044663 | 0.010976 | 0.092953 |
| GSM4058756 | NP | 0.012424 | 0.039346 | 0.055881 |
| GSM4058757 | NP | 0.038239 | 0.026422 | 0.016917 |
| GSM4058758 | NP | 0.010053 | 0.007291 | 0.012309 |
| GSM4058759 | NP | 0 | 0.04605 | 0.045288 |
| GSM4058760 | NP | 0.017905 | 0.024234 | 0.016184 |
| GSM4058761 | NP | 0.014519 | 0.062946 | 0.055533 |
| GSM4058772 | CON | 0.002498 | 0.043827 | 0 |
| GSM4058773 | CON | 0.067355 | 0.090128 | 0 |
| GSM4058774 | CON | 0 | 0.00875 | 0 |
| GSM4058775 | CON | 0 | 0.023087 | 0.04259 |
| GSM4058776 | CON | 0 | 0.061314 | 0.039646 |
| GSM4058777 | NP | 0 | 0.020985 | 0.029503 |
| GSM4058778 | NP | 0 | 0.036335 | 0.072416 |
| GSM4058779 | NP | 0 | 0.01337 | 0.076952 |
| GSM4058780 | NP | 0 | 0.001862 | 0.104544 |
| GSM4058781 | NP | 0 | 0.018212 | 0.081037 |
| GSM4058782 | NP | 0 | 0 | 0.044538 |
| GSM4058783 | NP | 0.00544 | 0.0299 | 0.014045 |
| GSM4058784 | NP | 0 | 0.001297 | 0.057465 |
| GSM4058785 | NP | 0 | 0.002059 | 0.05579 |
| GSM4058786 | NP | 0 | 0.013341 | 0.014551 |
| GSM4058787 | NP | 0 | 0.00364 | 0.045785 |
| GSM4058788 | NP | 0.0067 | 0 | 0.035814 |
| GSM4058789 | NP | 0 | 0.048313 | 0.008838 |
| GSM4058790 | NP | 0.007402 | 0.021156 | 0 |
| GSM4058791 | NP | 0 | 0.010952 | 0.024338 |
| GSM4058792 | NP | 0 | 0.031043 | 0.051888 |
| GSM4058793 | NP | 0.014237 | 0.021475 | 0.007092 |
| GSM4058794 | NP | 0 | 0.027636 | 0.032621 |
| GSM4058795 | NP | 0.011478 | 0.061392 | 0.011487 |
| GSM4058796 | NP | 0 | 0.008729 | 0.004488 |
| GSM4058797 | NP | 0 | 0.054675 | 0.101354 |
| GSM4058798 | NP | 0.006351 | 0.087506 | 0.030708 |
| GSM4058799 | NP | 0.036031 | 0.008063 | 0.009682 |
| GSM4058800 | NP | 0 | 0.012362 | 0.088411 |
| GSM4058801 | NP | 0 | 0.006665 | 0.259462 |
| GSM4058825 | NP | 0.025827 | 0.015126 | 0.08231 |
| GSM4058826 | NP | 0.015945 | 0.028504 | 0.017263 |
| GSM4058827 | NP | 0 | 0 | 0.185592 |
| GSM4058828 | NP | 0 | 0.051127 | 0.078742 |
| GSM4058829 | NP | 0 | 0.003658 | 0.067115 |
| GSM4058830 | NP | 0.02288 | 0.063109 | 0.100127 |
| GSM4058831 | NP | 0 | 0.033191 | 0.04456 |
| GSM4058832 | CON | 0 | 0.003362 | 0.021032 |
| GSM4058833 | CON | 0.013053 | 0.002402 | 0 |
| GSM4058834 | CON | 0 | 0.008206 | 0.033095 |
| GSM4058835 | CON | 0 | 0.012381 | 0 |
| GSM4058836 | CON | 0 | 0.01066 | 0 |
| GSM4058837 | CON | 0 | 0 | 0 |
| GSM4058838 | CON | 0.005226 | 0.017484 | 0.025381 |
| GSM4058839 | CON | 0 | 0.030685 | 0.028766 |
| GSM4058840 | CON | 0 | 0.030377 | 0.014498 |
| GSM4058841 | CON | 0 | 0.0019 | 0.024815 |
| GSM4058842 | CON | 0.005437 | 0 | 0 |
| GSM4058843 | CON | 0 | 0.042818 | 0.066551 |
| GSM4058844 | CON | 0.002481 | 0.332156 | 0 |
| GSM4058845 | CON | 0.00896 | 0.028075 | 0.018273 |
| GSM4058846 | CON | 0 | 0.004446 | 0.009959 |
| GSM4058847 | CON | 0 | 0.013106 | 0.010841 |
| GSM4058848 | CON | 0 | 0.013598 | 0 |
| GSM4058849 | CON | 0 | 0.072438 | 0.007319 |
| GSM4058850 | CON | 0.006724 | 0 | 0 |
| GSM4058851 | CON | 0.042351 | 0.029483 | 0 |
| GSM4058852 | CON | 0 | 0.013141 | 0.032044 |
| GSM4058853 | CON | 0 | 0.013778 | 0.005852 |
| GSM4058854 | CON | 0.024607 | 0.070066 | 0 |
| GSM5412745 | CON | 0.162696 | 0.068273 | 0 |
| GSM5412746 | CON | 0.015619 | 0.184335 | 0 |
| GSM5412747 | CON | 0 | 0.026392 | 0.001509 |
| GSM5412748 | CON | 0.037483 | 0.111289 | 0 |
| GSM5412749 | CON | 0.023004 | 0.046019 | 0 |
| GSM5412750 | CON | 0.048964 | 0.020451 | 0 |
| GSM5412751 | CON | 0.005514 | 0.033986 | 0 |
| GSM5412752 | NP | 0 | 0.011328 | 0 |
| GSM5412753 | NP | 0 | 0.052621 | 0.012081 |
| GSM5412754 | NP | 0.009252 | 0.089918 | 0 |
| GSM5412755 | NP | 0 | 0.099907 | 0.008345 |
| GSM5412756 | NP | 0 | 0.036816 | 0.028575 |
| GSM5412757 | NP | 0 | 0.005496 | 0.055162 |
| GSM5412758 | NP | 0 | 0 | 0.150095 |
| GSM5412759 | NP | 0.014955 | 0.003854 | 0 |
| GSM5412760 | NP | 0 | 0 | 0.035908 |
| GSM5412761 | NP | 0 | 0.00766 | 0.219281 |
| GSM5412762 | NP | 0 | 0.004863 | 0.096582 |
| GSM5412763 | NP | 0 | 0 | 0.116607 |
| GSM5412764 | NP | 0 | 0.039614 | 0.006581 |
| GSM5412765 | NP | 0 | 0.007973 | 0.091083 |
| GSM5412766 | NP | 0 | 0.008602 | 0.046821 |
| GSM5412767 | NP | 0 | 0.015571 | 0.054444 |
| GSM5412768 | NP | 0 | 0.005365 | 0.10344 |

**Supplementary Table 2.** Ninety-two M2 macrophage-associated DEGs in CRSwNP

| **Genes** | **logFC** | **AveExpr** | **P.Value** | **adj.P.Val** |  |
| --- | --- | --- | --- | --- | --- |
| SCG2 | 2.726341 | 2.805047 | 1.37E-17 | 4.82E-15 | UP |
| F13A1 | 2.409975 | 4.370596 | 4.90E-14 | 4.28E-12 | UP |
| CD163 | 2.259848 | 2.812404 | 3.37E-16 | 5.92E-14 | UP |
| COL10A1 | 2.247006 | 2.67984 | 2.55E-15 | 3.38E-13 | UP |
| LAMP5 | 2.039648 | 4.030945 | 3.55E-11 | 1.29E-09 | UP |
| C1QB | 1.99228 | 4.28871 | 5.15E-13 | 3.26E-11 | UP |
| HMOX1 | 1.880872 | 2.994019 | 6.79E-19 | 6.45E-16 | UP |
| VSIG4 | 1.85052 | 3.54858 | 8.72E-12 | 3.80E-10 | UP |
| C3 | 1.82904 | 5.107487 | 6.86E-12 | 3.12E-10 | UP |
| MS4A4A | 1.809477 | 2.891131 | 3.62E-13 | 2.47E-11 | UP |
| FOLR2 | 1.793412 | 3.722506 | 1.61E-10 | 4.95E-09 | UP |
| C1QA | 1.759658 | 4.358523 | 2.10E-09 | 4.46E-08 | UP |
| CYBB | 1.758516 | 3.254486 | 1.08E-13 | 8.39E-12 | UP |
| TRBD1 | 1.757678 | 2.78836 | 8.07E-05 | 0.00041 | UP |
| TYROBP | 1.722044 | 3.163061 | 1.24E-14 | 1.31E-12 | UP |
| C1QC | 1.685526 | 4.761236 | 2.50E-11 | 9.62E-10 | UP |
| SPP1 | 1.67915 | 3.107728 | 1.25E-05 | 8.22E-05 | UP |
| FPR1 | 1.665275 | 2.584674 | 2.20E-11 | 8.57E-10 | UP |
| CSF1R | 1.657295 | 3.653179 | 1.94E-14 | 1.94E-12 | UP |
| TMEM176A | 1.646394 | 3.268547 | 6.96E-14 | 5.89E-12 | UP |
| COL1A1 | 1.58766 | 5.259168 | 3.04E-07 | 3.26E-06 | UP |
| CD4 | 1.582938 | 3.229094 | 1.24E-14 | 1.31E-12 | UP |
| MMP2 | 1.538861 | 4.326803 | 6.09E-13 | 3.75E-11 | UP |
| CLEC10A | 1.532814 | 2.229661 | 5.30E-10 | 1.37E-08 | UP |
| CPXM1 | 1.50267 | 4.697067 | 2.99E-10 | 8.32E-09 | UP |
| FCGR2A | 1.492395 | 2.712265 | 4.17E-13 | 2.75E-11 | UP |
| ITGB2 | 1.491072 | 3.387401 | 1.67E-12 | 8.95E-11 | UP |
| LUM | 1.49047 | 7.048684 | 1.46E-09 | 3.26E-08 | UP |
| HCK | 1.480161 | 2.891634 | 4.16E-12 | 2.00E-10 | UP |
| PTAFR | 1.47282 | 3.073746 | 4.84E-16 | 7.87E-14 | UP |
| C3AR1 | 1.470942 | 2.951018 | 9.70E-12 | 4.18E-10 | UP |
| LAPTM5 | 1.467375 | 5.344942 | 5.89E-13 | 3.67E-11 | UP |
| ADAM8 | 1.462107 | 2.635545 | 5.43E-15 | 6.50E-13 | UP |
| RARRES1 | 1.454568 | 6.371808 | 1.82E-05 | 0.000114 | UP |
| MS4A6A | 1.419598 | 3.175071 | 1.58E-12 | 8.57E-11 | UP |
| ALOX5 | 1.408923 | 2.936917 | 9.83E-14 | 7.82E-12 | UP |
| TMEM176B | 1.40871 | 4.844592 | 8.22E-10 | 1.98E-08 | UP |
| NCF2 | 1.391758 | 3.541468 | 9.96E-11 | 3.20E-09 | UP |
| FCGR3A | 1.388114 | 3.467682 | 9.32E-09 | 1.61E-07 | UP |
| FPR3 | 1.37581 | 2.22112 | 1.58E-12 | 8.57E-11 | UP |
| ALOX5AP | 1.355926 | 2.844966 | 1.48E-09 | 3.30E-08 | UP |
| FCER1G | 1.343221 | 3.43663 | 1.70E-10 | 5.18E-09 | UP |
| LYVE1 | 1.327071 | 2.020441 | 8.78E-07 | 8.23E-06 | UP |
| MPEG1 | 1.324574 | 3.301707 | 6.20E-11 | 2.09E-09 | UP |
| COL8A1 | 1.31515 | 2.587482 | 2.31E-09 | 4.83E-08 | UP |
| CD300A | 1.30372 | 2.251133 | 2.20E-11 | 8.57E-10 | UP |
| CD86 | 1.301008 | 2.046152 | 3.56E-11 | 1.29E-09 | UP |
| SELPLG | 1.288828 | 3.929338 | 3.44E-10 | 9.39E-09 | UP |
| CD53 | 1.280725 | 4.801571 | 9.08E-09 | 1.57E-07 | UP |
| SAMSN1 | 1.278955 | 2.947926 | 3.53E-13 | 2.41E-11 | UP |
| RAC2 | 1.272532 | 3.880148 | 1.92E-09 | 4.15E-08 | UP |
| SRGN | 1.263753 | 6.407927 | 2.58E-10 | 7.37E-09 | UP |
| COL1A2 | 1.259398 | 5.786159 | 1.70E-06 | 1.45E-05 | UP |
| SLA | 1.253606 | 2.334922 | 3.17E-12 | 1.57E-10 | UP |
| MRC1 | 1.240509 | 2.321654 | 3.74E-08 | 5.20E-07 | UP |
| TGM2 | 1.214884 | 5.159929 | 5.05E-08 | 6.82E-07 | UP |
| MS4A7 | 1.173039 | 2.565287 | 5.66E-10 | 1.46E-08 | UP |
| PLEK | 1.170014 | 3.449428 | 2.26E-07 | 2.54E-06 | UP |
| AIF1 | 1.160329 | 2.39235 | 3.73E-10 | 1.00E-08 | UP |
| SERPINA1 | 1.156482 | 3.575768 | 5.86E-07 | 5.78E-06 | UP |
| CST7 | 1.152372 | 2.607681 | 9.76E-08 | 1.21E-06 | UP |
| THBS2 | 1.143038 | 2.950117 | 1.08E-06 | 9.88E-06 | UP |
| SPON1 | 1.141413 | 3.015468 | 1.56E-09 | 3.46E-08 | UP |
| CD37 | 1.136772 | 2.268241 | 1.46E-08 | 2.35E-07 | UP |
| CFI | 1.130091 | 3.504542 | 5.37E-11 | 1.86E-09 | UP |
| THEMIS2 | 1.124133 | 2.487511 | 1.13E-08 | 1.90E-07 | UP |
| CCR1 | 1.123893 | 2.155435 | 1.13E-07 | 1.37E-06 | UP |
| CXCR4 | 1.117187 | 4.854735 | 2.05E-08 | 3.14E-07 | UP |
| SASH3 | 1.117164 | 3.50119 | 4.72E-08 | 6.44E-07 | UP |
| CPVL | 1.094703 | 3.976285 | 2.20E-07 | 2.48E-06 | UP |
| SLCO2B1 | 1.093559 | 2.449698 | 6.54E-10 | 1.66E-08 | UP |
| MS4A1 | 1.088453 | 2.019473 | 0.000337 | 0.001398 | UP |
| LRRC25 | 1.087601 | 2.223143 | 8.71E-07 | 8.18E-06 | UP |
| ARHGAP30 | 1.084797 | 3.069803 | 9.23E-10 | 2.19E-08 | UP |
| SULF1 | 1.081437 | 3.418386 | 6.46E-09 | 1.17E-07 | UP |
| IL10RA | 1.079913 | 2.598651 | 8.49E-09 | 1.49E-07 | UP |
| VNN2 | 1.078718 | 2.282773 | 1.84E-07 | 2.12E-06 | UP |
| SAA2 | 1.074215 | 2.062648 | 0.000457 | 0.001825 | UP |
| CHI3L1 | 1.054716 | 2.33561 | 0.000146 | 0.000685 | UP |
| DOK2 | 1.045861 | 2.650339 | 1.50E-06 | 1.30E-05 | UP |
| GPSM3 | 1.040399 | 3.116342 | 1.42E-10 | 4.41E-09 | UP |
| CD1C | 1.029781 | 2.242011 | 0.000116 | 0.000562 | UP |
| TGFBI | 1.027673 | 3.977962 | 6.92E-10 | 1.74E-08 | UP |
| ARHGAP4 | 1.025584 | 2.38474 | 1.69E-11 | 6.79E-10 | UP |
| NPL | 1.023666 | 2.511862 | 1.31E-11 | 5.40E-10 | UP |
| PDGFRA | 1.022789 | 2.746753 | 7.47E-09 | 1.32E-07 | UP |
| NCF4 | 1.020123 | 2.90577 | 7.63E-07 | 7.26E-06 | UP |
| ARRB2 | 1.018391 | 2.450537 | 2.99E-17 | 8.62E-15 | UP |
| HCLS1 | 1.014816 | 4.098811 | 8.09E-10 | 1.96E-08 | UP |
| EVI2B | 1.014727 | 3.574702 | 2.12E-07 | 2.41E-06 | UP |
| FAM3B | -1.02592 | 3.151665 | 1.69E-07 | 1.98E-06 | DOWN |
| KLK11 | -1.07488 | 3.456407 | 1.19E-05 | 7.96E-05 | DOWN |

**Supplementary Table 3.** GO Functional enrichment analysis of M2 macrophage-associated DEGs. (Top 50 terms with the smallest adjusted P value in each category was shown)

| **Category** | **ID** | **Description** | **p.adjust** | **geneID** | **Count** |
| --- | --- | --- | --- | --- | --- |
| BP | GO:0050900 | leukocyte migration | 1.8151E-09 | GPSM3/CD300A/SCG2/CXCR4/SELPLG/  RAC2/PTAFR/ITGB2/HMOX1/HCK/  FCER1G/CSF1R/CCR1/C3AR1/ALOX5/  AIF1/ADAM8 | 17 |
| BP | GO:0001819 | positive regulation of cytokine production | 3.6179E-09 | GPSM3/SASH3/SULF1/LAPTM5/TYROBP/  PTAFR/LUM/HMOX1/FCER1G/CYBB/  CSF1R/CHI3L1/CD86/CD4/C3AR1/C3/  AIF1/ADAM8 | 18 |
| BP | GO:0002253 | activation of immune response | 9.6139E-09 | VSIG4/CD300A/THEMIS2/LAPTM5/  TYROBP/CFI/HCK/FPR3/FPR1/FCER1G/  MS4A1/C3AR1/C3/C1QC/C1QB/C1QA | 16 |
| BP | GO:0060326 | cell chemotaxis | 7.2128E-08 | GPSM3/SCG2/CXCR4/RAC2/PDGFRA/  ITGB2/FCER1G/CSF1R/CCR1/C3AR1/  ARRB2/ALOX5/AIF1/ADAM8 | 14 |
| BP | GO:0030595 | leukocyte chemotaxis | 2.5396E-07 | GPSM3/SCG2/CXCR4/RAC2/ITGB2/  FCER1G/CSF1R/CCR1/C3AR1/ALOX5/  AIF1/ADAM8 | 12 |
| BP | GO:0002443 | leukocyte mediated immunity | 4.9388E-07 | SASH3/CD300A/TYROBP/RAC2/PTAFR/  ITGB2/CFI/HMOX1/FCER1G/CD1C/C3/  C1QC/C1QB/C1QA/ARRB2 | 15 |
| BP | GO:0002697 | regulation of immune effector process | 1.4243E-06 | SASH3/VSIG4/CD300A/LAPTM5/TYROBP/  RAC2/PTAFR/ITGB2/HMOX1/CD86/CD1C/  C3/ARRB2 | 13 |
| BP | GO:0002274 | myeloid leukocyte activation | 1.4261E-06 | VSIG4/CD300A/CST7/TYROBP/RAC2/  PTAFR/ITGB2/HMOX1/FCER1G/C1QA/  AIF1 | 11 |
| BP | GO:0002886 | regulation of myeloid leukocyte mediated immunity | 1.4261E-06 | CD300A/TYROBP/RAC2/PTAFR/ITGB2/  HMOX1/C3 | 7 |
| BP | GO:0032103 | positive regulation of response to external stimulus | 1.8005E-06 | GPSM3/SCG2/CXCR4/TYROBP/TGM2/  RAC2/HCK/CSF1R/CCR1/C3AR1/C3/  ALOX5AP/AIF1/ADAM8 | 14 |
| BP | GO:0002699 | positive regulation of immune effector process | 1.9303E-06 | SASH3/CD300A/LAPTM5/TYROBP/  RAC2/PTAFR/ITGB2/HMOX1/CD86/  CD1C/C3 | 11 |
| BP | GO:0007159 | leukocyte cell-cell adhesion | 2.432E-06 | SASH3/VSIG4/CD300A/LAPTM5/SELPLG/  RAC2/PTAFR/ITGB2/CD86/CD4/ALOX5/  AIF1/ADAM8 | 13 |
| BP | GO:0002685 | regulation of leukocyte migration | 6.7137E-06 | GPSM3/CD300A/RAC2/PTAFR/HMOX1/  CSF1R/CCR1/C3AR1/AIF1/ADAM8 | 10 |
| BP | GO:1902105 | regulation of leukocyte differentiation | 8.8231E-06 | TMEM176A/SASH3/TMEM176B/TYROBP/  HCLS1/EVI2B/CCR1/CD86/CD4/C1QC/  ADAM8 | 11 |
| BP | GO:0097529 | myeloid leukocyte migration | 9.0132E-06 | CD300A/SCG2/RAC2/ITGB2/FCER1G/  CSF1R/CCR1/C3AR1/AIF1/ADAM8 | 10 |
| BP | GO:0002703 | regulation of leukocyte mediated immunity | 1.0763E-05 | SASH3/CD300A/TYROBP/RAC2/PTAFR/  ITGB2/HMOX1/CD1C/C3/ARRB2 | 10 |
| BP | GO:0002683 | negative regulation of immune system process | 1.0763E-05 | SAMSN1/TMEM176A/TMEM176B/  VSIG4/CD300A/CST7/LAPTM5/TYROBP/  HMOX1/HCK/CD86/C1QC/ARRB2 | 13 |
| BP | GO:0098883 | synapse pruning | 1.4146E-05 | C3/C1QC/C1QB/C1QA | 4 |
| BP | GO:0006909 | phagocytosis | 1.7636E-05 | CD300A/TYROBP/TGM2/RAC2/NCF4/  NCF2/ITGB2/HCK/FCER1G/C3/AIF1 | 11 |
| BP | GO:0002687 | positive regulation of leukocyte migration | 1.8711E-05 | GPSM3/RAC2/PTAFR/CSF1R/CCR1/  C3AR1/AIF1/ADAM8 | 8 |
| BP | GO:0002275 | myeloid cell activation involved in immune response | 1.8711E-05 | CD300A/TYROBP/RAC2/PTAFR/ITGB2/  HMOX1/FCER1G | 7 |
| BP | GO:0050921 | positive regulation of chemotaxis | 2.4804E-05 | GPSM3/SCG2/CXCR4/RAC2/CSF1R/CCR1/  C3AR1/AIF1 | 8 |
| BP | GO:0002444 | myeloid leukocyte mediated immunity | 3.0496E-05 | CD300A/TYROBP/RAC2/PTAFR/ITGB2/  HMOX1/C3 | 7 |
| BP | GO:0097530 | granulocyte migration | 3.1905E-05 | CD300A/SCG2/RAC2/ITGB2/FCER1G/  CSF1R/C3AR1/ADAM8 | 8 |
| BP | GO:1903037 | regulation of leukocyte cell-cell adhesion | 3.1905E-05 | SASH3/VSIG4/CD300A/LAPTM5/PTAFR/  ITGB2/CD86/CD4/ALOX5/AIF1/ADAM8 | 11 |
| BP | GO:0050866 | negative regulation of cell activation | 3.9857E-05 | SAMSN1/VSIG4/CD300A/CST7/LAPTM5/  TYROBP/PDGFRA/HMOX1/CD86 | 9 |
| BP | GO:0042119 | neutrophil activation | 4.2754E-05 | CD300A/TYROBP/PTAFR/ITGB2/FCER1G | 5 |
| BP | GO:1902107 | positive regulation of leukocyte differentiation | 4.2754E-05 | SASH3/TYROBP/HCLS1/EVI2B/CCR1/  CD86/CD4/ADAM8 | 8 |
| BP | GO:1903708 | positive regulation of hemopoiesis | 4.2754E-05 | SASH3/TYROBP/HCLS1/EVI2B/CCR1/  CD86/CD4/ADAM8 | 8 |
| BP | GO:0045730 | respiratory burst | 4.5338E-05 | RAC2/NCF4/NCF2/HCK/CYBB | 5 |
| BP | GO:0050920 | regulation of chemotaxis | 5.4127E-05 | GPSM3/SCG2/CXCR4/RAC2/PDGFRA/  CSF1R/CCR1/C3AR1/AIF1 | 9 |
| BP | GO:0002429 | immune response-activating cell surface receptor signaling pathway | 5.4127E-05 | CD300A/THEMIS2/LAPTM5/TYROBP/  HCK/FPR3/FPR1/FCER1G/MS4A1/C3AR1 | 10 |
| BP | GO:0002757 | immune response-activating signal transduction | 5.4127E-05 | CD300A/THEMIS2/LAPTM5/TYROBP/  HCK/FPR3/FPR1/FCER1G/MS4A1/C3AR1 | 10 |
| BP | GO:1903706 | regulation of hemopoiesis | 5.5908E-05 | TMEM176A/SASH3/TMEM176B/TYROBP/  HCLS1/EVI2B/CCR1/CD86/CD4/C1QC/  ADAM8 | 11 |
| BP | GO:0043299 | leukocyte degranulation | 5.8275E-05 | CD300A/RAC2/PTAFR/ITGB2/HMOX1/  HCK | 6 |
| BP | GO:0002283 | neutrophil activation involved in immune response | 6.3976E-05 | TYROBP/PTAFR/ITGB2/FCER1G | 4 |
| BP | GO:0030198 | extracellular matrix organization | 6.5523E-05 | SULF1/TGFBI/PDGFRA/MMP2/LUM/  COL10A1/COL8A1/COL1A2/COL1A1/  ADAM8 | 10 |
| BP | GO:0043062 | extracellular structure organization | 6.5736E-05 | SULF1/TGFBI/PDGFRA/MMP2/LUM/  COL10A1/COL8A1/COL1A2/COL1A1/  ADAM8 | 10 |
| BP | GO:0045229 | external encapsulating structure organization | 6.7976E-05 | SULF1/TGFBI/PDGFRA/MMP2/LUM/  COL10A1/COL8A1/COL1A2/COL1A1/  ADAM8 | 10 |
| BP | GO:0002888 | positive regulation of myeloid leukocyte mediated immunity | 7.1672E-05 | TYROBP/PTAFR/ITGB2/C3 | 4 |
| BP | GO:0036230 | granulocyte activation | 7.1672E-05 | CD300A/TYROBP/PTAFR/ITGB2/  FCER1G | 5 |
| BP | GO:0042554 | superoxide anion generation | 7.8648E-05 | TYROBP/NCF4/NCF2/ITGB2/CYBB | 5 |
| BP | GO:0002768 | immune response-regulating cell surface receptor signaling pathway | 8.4846E-05 | CD300A/THEMIS2/LAPTM5/TYROBP/  HCK/FPR3/FPR1/FCER1G/MS4A1/C3AR1 | 10 |
| BP | GO:0043300 | regulation of leukocyte degranulation | 9.4068E-05 | CD300A/RAC2/PTAFR/ITGB2/HMOX1 | 5 |
| BP | GO:0150146 | cell junction disassembly | 9.8323E-05 | C3/C1QC/C1QB/C1QA | 4 |
| BP | GO:0001774 | microglial cell activation | 9.8323E-05 | CST7/TYROBP/ITGB2/C1QA/AIF1 | 5 |
| BP | GO:0002695 | negative regulation of leukocyte activation | 9.8323E-05 | SAMSN1/VSIG4/CD300A/CST7/LAPTM5/  TYROBP/HMOX1/CD86 | 8 |
| BP | GO:0002690 | positive regulation of leukocyte chemotaxis | 0.00018802 | GPSM3/RAC2/CSF1R/CCR1/C3AR1/AIF1 | 6 |
| BP | GO:0002366 | leukocyte activation involved in immune response | 0.00019546 | CD300A/TYROBP/RAC2/PTAFR/ITGB2/  HMOX1/FCER1G/CD86/CD1C | 9 |
| BP | GO:0002263 | cell activation involved in immune response | 0.00021529 | CD300A/TYROBP/RAC2/PTAFR/ITGB2/  HMOX1/FCER1G/CD86/CD1C | 9 |
| MF | GO:0005201 | extracellular matrix structural constituent | 1.7139E-06 | SPON1/THBS2/TGFBI/LUM/COL10A1/  COL8A1/COL1A2/COL1A1/CHI3L1/CD4 | 10 |
| MF | GO:0140375 | immune receptor activity | 4.0687E-05 | CXCR4/IL10RA/FPR3/FPR1/FCER1G/  CCR1/CD4/C3AR1 | 8 |
| MF | GO:0019865 | immunoglobulin binding | 0.00025873 | FCGR3A/FCGR2A/FCER1G/MS4A1 | 4 |
| MF | GO:0019864 | IgG binding | 0.00065907 | FCGR3A/FCGR2A/FCER1G | 3 |
| MF | GO:0048407 | platelet-derived growth factor binding | 0.00065907 | PDGFRA/COL1A2/COL1A1 | 3 |
| MF | GO:0004875 | complement receptor activity | 0.00071274 | FPR3/FPR1/C3AR1 | 3 |
| MF | GO:0001618 | virus receptor activity | 0.00071274 | CXCR4/SELPLG/MRC1/CD86/CD4 | 5 |
| MF | GO:0038024 | cargo receptor activity | 0.00071274 | CD163/MRC1/ITGB2/CFI/FOLR2 | 5 |
| MF | GO:0140272 | exogenous protein binding | 0.00071274 | CXCR4/SELPLG/MRC1/CD86/CD4 | 5 |
| MF | GO:0030020 | extracellular matrix structural constituent conferring tensile strength | 0.00083182 | COL10A1/COL8A1/COL1A2/COL1A1 | 4 |
| MF | GO:0019955 | cytokine binding | 0.00943268 | CXCR4/IL10RA/CSF1R/CCR1/CD4 | 5 |
| MF | GO:0016175 | superoxide-generating NAD(P)H oxidase activity | 0.01497937 | NCF2/CYBB | 2 |
| MF | GO:0016176 | superoxide-generating NADPH oxidase activator activity | 0.01497937 | NCF4/NCF2 | 2 |
| MF | GO:0004896 | cytokine receptor activity | 0.01662022 | CXCR4/IL10RA/CCR1/CD4 | 4 |
| MF | GO:0015026 | coreceptor activity | 0.0202922 | CXCR4/CD86/CD4 | 3 |
| MF | GO:0008236 | serine-type peptidase activity | 0.02465197 | CPVL/KLK11/MMP2/CFI/ADAM8 | 5 |
| MF | GO:0001846 | opsonin binding | 0.02465197 | VSIG4/ITGB2 | 2 |
| MF | GO:0050664 | oxidoreductase activity, acting on NAD(P)H, oxygen as acceptor | 0.02465197 | NCF2/CYBB | 2 |
| MF | GO:0017171 | serine hydrolase activity | 0.02465197 | CPVL/KLK11/MMP2/CFI/ADAM8 | 5 |
| MF | GO:0016755 | aminoacyltransferase activity | 0.03229924 | TGM2/F13A1 | 2 |
| MF | GO:0001848 | complement binding | 0.04115096 | VSIG4/ITGB2 | 2 |
| MF | GO:0019838 | growth factor binding | 0.04115096 | PDGFRA/IL10RA/COL1A2/COL1A1 | 4 |
| MF | GO:0008528 | G protein-coupled peptide receptor activity | 0.04207686 | CXCR4/FPR3/FPR1/CCR1 | 4 |
| MF | GO:0016493 | C-C chemokine receptor activity | 0.04207686 | CXCR4/CCR1 | 2 |
| MF | GO:0016702 | oxidoreductase activity, acting on single donors with incorporation of molecular oxygen, incorporation of two atoms of oxygen | 0.04207686 | ALOX5AP/ALOX5 | 2 |
| MF | GO:0016701 | oxidoreductase activity, acting on single donors with incorporation of molecular oxygen | 0.04237171 | ALOX5AP/ALOX5 | 2 |
| MF | GO:0019957 | C-C chemokine binding | 0.04237171 | CXCR4/CCR1 | 2 |
| MF | GO:0001653 | peptide receptor activity | 0.04274863 | CXCR4/FPR3/FPR1/CCR1 | 4 |
| MF | GO:0051019 | mitogen-activated protein kinase binding | 0.04274863 | PTAFR/ARRB2 | 2 |
| MF | GO:0001637 | G protein-coupled chemoattractant receptor activity | 0.04319061 | CXCR4/CCR1 | 2 |
| MF | GO:0004950 | chemokine receptor activity | 0.04319061 | CXCR4/CCR1 | 2 |
| MF | GO:0023026 | MHC class II protein complex binding | 0.04504992 | MS4A1/CD4 | 2 |
| MF | GO:0004252 | serine-type endopeptidase activity | 0.05704129 | KLK11/MMP2/CFI/ADAM8 | 4 |
| MF | GO:0004866 | endopeptidase inhibitor activity | 0.06083145 | CST7/RARRES1/SERPINA1/C3 | 4 |
| MF | GO:0019956 | chemokine binding | 0.06083145 | CXCR4/CCR1 | 2 |
| MF | GO:0030414 | peptidase inhibitor activity | 0.06677356 | CST7/RARRES1/SERPINA1/C3 | 4 |
| MF | GO:0023023 | MHC protein complex binding | 0.0680331 | MS4A1/CD4 | 2 |
| MF | GO:0061135 | endopeptidase regulator activity | 0.07156613 | CST7/RARRES1/SERPINA1/C3 | 4 |
| MF | GO:0008047 | enzyme activator activity | 0.07894725 | NCF4/NCF2/ALOX5AP | 3 |
| MF | GO:0004180 | carboxypeptidase activity | 0.08829523 | CPXM1/CPVL | 2 |
| MF | GO:0001784 | phosphotyrosine residue binding | 0.08996926 | SAMSN1/HCK | 2 |
| MF | GO:0005044 | scavenger receptor activity | 0.09816468 | CD163/CFI | 2 |
| MF | GO:0004714 | transmembrane receptor protein tyrosine kinase activity | 0.09816468 | PDGFRA/HCK/CSF1R | 3 |
| MF | GO:0061134 | peptidase regulator activity | 0.10806161 | CST7/RARRES1/SERPINA1/C3 | 4 |
| MF | GO:0002020 | protease binding | 0.11654727 | SERPINA1/COL1A2/COL1A1 | 3 |
| MF | GO:0004713 | protein tyrosine kinase activity | 0.11654727 | PDGFRA/HCK/CSF1R | 3 |
| MF | GO:0045309 | protein phosphorylated amino acid binding | 0.12062364 | SAMSN1/HCK | 2 |
| MF | GO:0050840 | extracellular matrix binding | 0.12062364 | TGFBI/SPP1 | 2 |
| MF | GO:0019199 | transmembrane receptor protein kinase activity | 0.12407626 | PDGFRA/HCK/CSF1R | 3 |
| MF | GO:0005178 | integrin binding | 0.12407626 | TGFBI/SPP1/ITGB2 | 3 |
| CC | GO:0062023 | collagen-containing extracellular matrix | 1.963E-08 | SULF1/SPON1/THBS2/TGM2/TGFBI/  SERPINA1/MMP2/LUM/F13A1/COL10A1/  COL8A1/COL1A2/COL1A1/C1QC/C1QB/  C1QA | 16 |
| CC | GO:0005581 | collagen trimer | 5.7046E-07 | LUM/COL10A1/COL8A1/COL1A2/COL1A1/  C1QC/C1QB/C1QA | 8 |
| CC | GO:0070821 | tertiary granule membrane | 2.5654E-06 | CD300A/PTAFR/ITGB2/FCER1G/CYBB/  CD53/ADAM8 | 7 |
| CC | GO:0030667 | secretory granule membrane | 8.8769E-06 | CD300A/TYROBP/PTAFR/ITGB2/FPR1/  FCGR2A/FCER1G/CYBB/CD53/C3AR1/  ADAM8 | 11 |
| CC | GO:0009897 | external side of plasma membrane | 1.9561E-05 | CD163/CXCR4/PDGFRA/ITGB2/FOLR2/  FCGR3A/FCER1G/CCR1/CD86/MS4A1/  CD4/CD1C | 12 |
| CC | GO:0070820 | tertiary granule | 2.6449E-05 | CD300A/PTAFR/ITGB2/FPR1/FCER1G/  CYBB/CD53/ADAM8 | 8 |
| CC | GO:0005788 | endoplasmic reticulum lumen | 4.6442E-05 | SPON1/SCG2/SPP1/SERPINA1/COL10A1/  COL8A1/COL1A2/COL1A1/CD4/C3 | 10 |
| CC | GO:0098644 | complex of collagen trimers | 4.8468E-05 | LUM/COL8A1/COL1A2/COL1A1 | 4 |
| CC | GO:0101003 | ficolin-1-rich granule membrane | 0.00016654 | CD300A/ITGB2/FPR1/FCER1G/ADAM8 | 5 |
| CC | GO:0044853 | plasma membrane raft | 0.00026895 | MS4A4A/SELPLG/ITGB2/HMOX1/  HCK/MS4A1 | 6 |
| CC | GO:0005583 | fibrillar collagen trimer | 0.00026895 | LUM/COL1A2/COL1A1 | 3 |
| CC | GO:0098643 | banded collagen fibril | 0.00026895 | LUM/COL1A2/COL1A1 | 3 |
| CC | GO:0101002 | ficolin-1-rich granule | 0.00031128 | CD300A/SERPINA1/ITGB2/FPR1/  FCER1G/ALOX5/ADAM8 | 7 |
| CC | GO:0030139 | endocytic vesicle | 0.00031271 | MPEG1/CD163/RAC2/NCF4/NCF2/  CYBB/CD4/ARRB2/ADAM8 | 9 |
| CC | GO:0043020 | NADPH oxidase complex | 0.00035357 | NCF4/NCF2/CYBB | 3 |
| CC | GO:0045335 | phagocytic vesicle | 0.00043273 | MPEG1/RAC2/NCF4/NCF2/CYBB/  ADAM8 | 6 |
| CC | GO:0005767 | secondary lysosome | 0.00057687 | NCF4/NCF2/ADAM8 | 3 |
| CC | GO:0035579 | specific granule membrane | 0.00058255 | ITGB2/CYBB/CD53/C3AR1/ADAM8 | 5 |
| CC | GO:0042581 | specific granule | 0.00086189 | ITGB2/CYBB/CHI3L1/CD53/C3AR1/  ADAM8 | 6 |
| CC | GO:0045121 | membrane raft | 0.00130277 | MS4A4A/SULF1/SELPLG/ITGB2/  HMOX1/HCK/MS4A1/CD4 | 8 |
| CC | GO:0098857 | membrane microdomain | 0.00130277 | MS4A4A/SULF1/SELPLG/ITGB2/  HMOX1/HCK/MS4A1/CD4 | 8 |
| CC | GO:0031091 | platelet alpha granule | 0.00612277 | THBS2/SRGN/SERPINA1/F13A1 | 4 |
| CC | GO:0005884 | actin filament | 0.01347026 | RAC2/HCLS1/HCK/AIF1 | 4 |
| CC | GO:0031093 | platelet alpha granule lumen | 0.02429168 | SRGN/SERPINA1/F13A1 | 3 |
| CC | GO:0034774 | secretory granule lumen | 0.02484966 | SRGN/SERPINA1/F13A1/CHI3L1/C3/  ALOX5 | 6 |
| CC | GO:0060205 | cytoplasmic vesicle lumen | 0.02484966 | SRGN/SERPINA1/F13A1/CHI3L1/C3/  ALOX5 | 6 |
| CC | GO:0031983 | vesicle lumen | 0.02484966 | SRGN/SERPINA1/F13A1/CHI3L1/C3/  ALOX5 | 6 |
| CC | GO:0072562 | blood microparticle | 0.0268804 | F13A1/C3/C1QC/C1QB | 4 |
| CC | GO:0031045 | dense core granule | 0.03684018 | SCG2/ADAM8 | 2 |
| CC | GO:0005604 | basement membrane | 0.05256736 | THBS2/TGFBI/COL8A1 | 3 |
| CC | GO:0030666 | endocytic vesicle membrane | 0.063427 | CD163/RAC2/CYBB/CD4 | 4 |
| CC | GO:0031252 | cell leading edge | 0.06879221 | SAMSN1/LAMP5/CXCR4/RAC2/PLEK/  AIF1 | 6 |
| CC | GO:1990204 | oxidoreductase complex | 0.07225036 | NCF4/NCF2/CYBB | 3 |
| CC | GO:0001772 | immunological synapse | 0.08042526 | CD53/CD37 | 2 |
| CC | GO:0035577 | azurophil granule membrane | 0.13086255 | FPR1/C3AR1 | 2 |
| CC | GO:0005766 | primary lysosome | 0.14912872 | FPR1/C3AR1/C3 | 3 |
| CC | GO:0042582 | azurophil granule | 0.14912872 | FPR1/C3AR1/C3 | 3 |
| CC | GO:0005925 | focal adhesion | 0.18050303 | TGM2/SLA/RAC2/ITGB2/HCK | 5 |
| CC | GO:0030670 | phagocytic vesicle membrane | 0.18050303 | RAC2/CYBB | 2 |

**Supplementary Table 4.** KEGG pathway analysis of M2 macrophage-associated DEGs.

| **ID** | **Description** | **p.adjust** | **geneID** | **Count** |
| --- | --- | --- | --- | --- |
| hsa05150 | Staphylococcus aureus infection | 2.0094E-12 | SELPLG/PTAFR/ITGB2/CFI/FPR3/FPR1/  FCGR3A/FCGR2A/C3AR1/C3/C1QC/  C1QB/C1QA | 13 |
| hsa04610 | Complement and coagulation cascades | 6.1896E-09 | VSIG4/SERPINA1/ITGB2/CFI/F13A1/  C3AR1/C3/C1QC/C1QB/C1QA | 10 |
| hsa04613 | Neutrophil extracellular trap formation | 9.1454E-07 | SELPLG/RAC2/NCF4/NCF2/ITGB2/FPR3/  FPR1/FCGR3A/FCGR2A/CYBB/C3 | 11 |
| hsa04145 | Phagosome | 1.2196E-05 | THBS2/NCF4/NCF2/MRC1/ITGB2/FCGR3A/  FCGR2A/CYBB/C3 | 9 |
| hsa05140 | Leishmaniasis | 1.2196E-05 | NCF4/NCF2/ITGB2/FCGR3A/FCGR2A/CYBB/  C3 | 7 |
| hsa04670 | Leukocyte transendothelial migration | 0.00014525 | CXCR4/RAC2/NCF4/NCF2/MMP2/ITGB2/CYBB | 7 |
| hsa05322 | Systemic lupus erythematosus | 0.00039675 | FCGR3A/FCGR2A/CD86/C3/C1QC/C1QB/C1QA | 7 |
| hsa05171 | Coronavirus disease - COVID-19 | 0.00153332 | FCGR2A/F13A1/CYBB/C3AR1/C3/C1QC/C1QB/  C1QA | 8 |
| hsa05133 | Pertussis | 0.00159046 | ITGB2/C3/C1QC/C1QB/C1QA | 5 |
| hsa05152 | Tuberculosis | 0.00165789 | MRC1/ITGB2/IL10RA/FCGR3A/FCGR2A/  FCER1G/C3 | 7 |
| hsa04380 | Osteoclast differentiation | 0.00171748 | TYROBP/NCF4/NCF2/FCGR3A/FCGR2A/  CSF1R | 6 |
| hsa05415 | Diabetic cardiomyopathy | 0.00290496 | RAC2/NCF4/NCF2/MMP2/CYBB/  COL1A2/COL1A1 | 7 |
| hsa04640 | Hematopoietic cell lineage | 0.0038164 | CSF1R/CD37/MS4A1/CD4/CD1C | 5 |
| hsa04664 | Fc epsilon RI signaling pathway | 0.00795617 | RAC2/FCER1G/ALOX5AP/ALOX5 | 4 |
| hsa04650 | Natural killer cell mediated cytotoxicity | 0.01180219 | TYROBP/RAC2/ITGB2/FCGR3A/FCER1G | 5 |
| hsa05020 | Prion disease | 0.01221206 | RAC2/NCF4/NCF2/CYBB/C1QC/C1QB/  C1QA | 7 |
| hsa04510 | Focal adhesion | 0.01221206 | THBS2/SPP1/RAC2/PDGFRA/COL1A2/  COL1A1 | 6 |
| hsa04936 | Alcoholic liver disease | 0.01404693 | C3AR1/C3/C1QC/C1QB/C1QA | 5 |
| hsa04512 | ECM-receptor interaction | 0.01528251 | THBS2/SPP1/COL1A2/COL1A1 | 4 |
| hsa04666 | Fc gamma R-mediated phagocytosis | 0.02052774 | RAC2/HCK/FCGR3A/FCGR2A | 4 |
| hsa04061 | Viral protein interaction with cytokine and cytokine receptor | 0.02052774 | CXCR4/IL10RA/CSF1R/CCR1 | 4 |
| hsa04933 | AGE-RAGE signaling pathway in diabetic complications | 0.02052774 | MMP2/CYBB/COL1A2/COL1A1 | 4 |
| hsa05142 | Chagas disease | 0.02052774 | C3/C1QC/C1QB/C1QA | 4 |
| hsa05146 | Amoebiasis | 0.02052774 | ITGB2/COL1A2/COL1A1/CD1C | 4 |
| hsa04974 | Protein digestion and absorption | 0.02052774 | COL10A1/COL8A1/COL1A2/COL1A1 | 4 |
| hsa05416 | Viral myocarditis | 0.03128714 | RAC2/ITGB2/CD86 | 3 |
| hsa04062 | Chemokine signaling pathway | 0.03394153 | CXCR4/RAC2/HCK/CCR1/ARRB2 | 5 |
| hsa04611 | Platelet activation | 0.03525529 | FCGR2A/FCER1G/COL1A2/COL1A1 | 4 |
| hsa04926 | Relaxin signaling pathway | 0.03902634 | MMP2/COL1A2/COL1A1/ARRB2 | 4 |
| hsa05205 | Proteoglycans in cancer | 0.0399696 | MMP2/LUM/HCLS1/COL1A2/COL1A1 | 5 |
| hsa04015 | Rap1 signaling pathway | 0.0426545 | RAC2/PDGFRA/ITGB2/FPR1/CSF1R | 5 |
| hsa05418 | Fluid shear stress and atherosclerosis | 0.04566139 | RAC2/NCF2/MMP2/HMOX1 | 4 |
| hsa05163 | Human cytomegalovirus infection | 0.05283975 | CXCR4/RAC2/PDGFRA/IL10RA/CCR1 | 5 |
| hsa04514 | Cell adhesion molecules | 0.05432215 | SELPLG/ITGB2/CD86/CD4 | 4 |
| hsa04151 | PI3K-Akt signaling pathway | 0.08723302 | THBS2/SPP1/PDGFRA/CSF1R/COL1A2/  COL1A1 | 6 |
| hsa04216 | Ferroptosis | 0.09719708 | HMOX1/CYBB | 2 |
| hsa05167 | Kaposi sarcoma-associated herpesvirus infection | 0.11755982 | HCK/CCR1/CD86/C3 | 4 |
| hsa04672 | Intestinal immune network for IgA production | 0.12554558 | CXCR4/CD86 | 2 |
| hsa04060 | Cytokine-cytokine receptor interaction | 0.12554558 | CXCR4/IL10RA/CSF1R/CCR1/CD4 | 5 |
| hsa05144 | Malaria | 0.12603173 | THBS2/ITGB2 | 2 |
| hsa04810 | Regulation of actin cytoskeleton | 0.1519662 | CXCR4/RAC2/PDGFRA/ITGB2 | 4 |

**Supplementary Table 5.** Detail information of the M2 macrophage-associated genes involving in tissue remodeling.

| **Genes** | **Description** | **Function** |
| --- | --- | --- |
| **SPON1** | spondin 1 | an extracellular matrix structural constituent, involved in cell adhesion, located in extracellular space, Colocalizes with collagen-containing extracellular matrix. |
| **PDGFRA** | platelet derived growth factor receptor alpha | encodes a cell surface tyrosine kinase receptor for members of the platelet-derived growth factor family |
| **COL1A2** | collagen type I alpha 2 chain | encodes the pro-alpha2 chain of type I collagen, Type I is a fibril-forming collagen found in most connective tissues |
| **COL1A1** | collagen type I alpha 1 chain | encodes the pro-alpha1 chain of type I collagen, Type I is a fibril-forming collagen found in most connective tissues |
| **MMP2** | matrix metallopeptidase 2 | zinc-dependent enzymes capable of cleaving components of the extracellular matrix and molecules involved in signal transduction |
| **LUM** | lumican | a member of the small leucine-rich proteoglycan (SLRP) family, regulate collagen fibril organization and circumferential growth, corneal transparency, and epithelial cell migration and tissue repair |
| **THBS2** | thrombospondin 2 | a disulfide-linked homotrimeric glycoprotein that mediates cell-to-cell and cell-to-matrix interactions, a potent inhibitor of tumor growth and angiogenesis |
| **COL10A1** | collagen type X alpha 1 chain | encodes the alpha chain of type X collagen, a short chain collagen expressed by hypertrophic chondrocytes during endochondral ossification |
| **SERPINA1** | serpin family A member 1 | a serine protease inhibitor belonging to the serpin superfamily whose targets include elastase, plasmin, thrombin, trypsin, chymotrypsin, and plasminogen activator |
| **TGFBI** | transforming growth factor beta induced | encodes an RGD-containing protein that binds to type I, II and IV collagens, plays a role in cell-collagen interactions |
| **F13A1** | coagulation factor XIII A chain | encodes the coagulation factor XIII A subunit |
| **SULF1** | sulfatase 1 | encodes an extracellular heparan sulfate endosulfatase |
| **COL8A1** | collagen type VIII alpha 1 chain | encodes one of the two alpha chains of type VIII collagen, a short chain collagen and a major component of the basement membrane of the corneal endothelium |
| **TGM2** | transglutaminase 2 | enzymes that catalyze the crosslinking of proteins by epsilon-gamma glutamyl lysine isopeptide bonds |
| **C1QA** | complement C1q A chain | component of the serum complement system |
| **C1QB** | complement C1q B chain | component of the serum complement system |
| **C1QC** | complement C1q C chain | component of the serum complement system |
| **CD4** | CD4 molecule | a coreceptor with the T-cell receptor on the T lymphocyte to recognize antigens displayed by an antigen presenting cell in the context of class II MHC molecules |
| **CHI3L1** | chitinase 3 like 1 | Chitinases catalyze the hydrolysis of chitin, which is an abundant glycopolymer found in insect exoskeletons and fungal cell walls. The protein lacks chitinase activity and is secreted by activated macrophages, chondrocytes, neutrophils and synovial cells. The protein is thought to play a role in the process of inflammation and tissue remodeling |
| **ADAM8** | ADAM metallopeptidase domain 8 | This gene encodes a member of the ADAM (a disintegrin and metalloprotease domain) family. The protein encoded by this gene may be involved in cell adhesion during neurodegeneration, and it is thought to be a target for allergic respiratory diseases, including asthma. Alternative splicing results in multiple transcript variants. |

**Supplementary Table 6.** The top 20 hub genes calculated by 6 algorithms in CytoHubba

| **MNC** | **MCC** | **EPC** | **degree** | **closeness** | **radiality** |
| --- | --- | --- | --- | --- | --- |
| ITGB2 | TYROBP | FCGR3A | ITGB2 | ITGB2 | ITGB2 |
| TYROBP | FCGR3A | TYROBP | TYROBP | TYROBP | TYROBP |
| FCGR3A | CCR1 | ITGB2 | FCGR3A | FCGR3A | CD4 |
| C1QB | C3AR1 | C1QB | C1QB | C1QB | FCGR3A |
| CD4 | CYBB | CSF1R | CD4 | CD4 | C1QB |
| C1QA | AIF1 | IL10RA | C1QA | C1QA | C1QC |
| CD86 | C1QB | CD86 | CD53 | CYBB | C1QA |
| CD53 | C1QA | CYBB | CD86 | CD53 | CYBB |
| CYBB | FCER1G | C1QA | CYBB | CD86 | CSF1R |
| IL10RA | ITGB2 | LAPTM5 | CSF1R | CSF1R | PLEK |
| CSF1R | CD86 | CD4 | PLEK | PLEK | CCR1 |
| PLEK | IL10RA | FCER1G | IL10RA | C1QC | CD53 |
| LAPTM5 | HCK | CD53 | LAPTM5 | IL10RA | CD86 |
| HCK | CD4 | CCR1 | HCK | LAPTM5 | CD163 |
| CCR1 | CSF1R | C3AR1 | CCR1 | CCR1 | C3AR1 |
| C3AR1 | PLEK | PLEK | C1QC | HCK | AIF1 |
| CD163 | FCGR2A | HCK | C3AR1 | CD163 | NCF2 |
| C1QC | CD53 | FCGR2A | CD163 | C3AR1 | IL10RA |
| AIF1 | LAPTM5 | CD163 | AIF1 | AIF1 | LAPTM5 |
| FCER1G | CD163 | AIF1 | FCER1G | FCER1G | FCER1G |

**Supplementary Table 7.** Primers for RT-qPCR

| **Gene** | **Forward primer** | **Reverse primer** |
| --- | --- | --- |
| AIF1 | TCATGTCCCTGAAACGAATGC | TGGCTTTTCCTTTTCTCTCGC |
| C1QA | TCCCGGGAATTAAAGGCACC | TTCCTGGTTGGTGATGACCG |
| C1QB | TTCTGTGACTATGCCTACAACAC | GCCCAGTAGTGAGTTCTTGTC |
| C3AR1 | TGGCGTCTTTCTCTGCTGAG | TTCACTGTCCGCTGCATCTT |
| CCR1 | GACTATGACACGACCACAGAGT | CCAACCAGGCCAATGACAAATA |
| CD163 | TTTGTCAACTTGAGTCCCTTCAC | TCCCGCTACACTTGTTTTCAC |
| CD4 | CCTGGTAGTAGCCCCTCAGT | GGCCTTCTGGAAAGCTAGCA |
| CD53 | TCAACTTGCTCTTTTGGATCTGT | AGAACGACATAAGCAGACACTTG |
| CD86 | TTTGTGATGGCCTTCCTGCT | TGGAAACGTCGTACAGTTCTGT |
| CSF1R | GGGAATCCCAGTGATAGAGCC | TTGGAAGGTAGCGTTGTTGGT |
| CYBB | TGCCAGTCTGTCGAAATCTGC | ACTCGGGCATTCACACACC |
| FCER1G | TTCCAGCAGTGGTCTTGCTC | TGCCTTTCGCACTTGGATCT |
| FCGR3A | ATCTCCCAAAGGCTGTGGTG | GGGTGGAGAGGTTTGTCTGG |
| IL10RA | GAGATCCACAATGGCTTCATCC | TTCTCCAGAGGTTAGGAGGCT |
| ITGB2 | TGCGTCCTCTCTCAGGAGTG | GGTCCATGATGTCGTCAGCC |
| LAPTM5 | CTGCTTCAATGTCCGCATCG | CGATCAGTAGGCTCAGGCTG |
| PLEK | AAGAAGGGGAGCGTGTTCAAT | TCAGCGGGATCATTCCTTTGG |
| TYROBP | TGGTGCTGACAGTGCTCATT | GAGGTCGCTGTAGACATCCG |
